# Structural insights into assembly of the ribosomal nascent polypeptide exit tunnel

**DOI:** 10.1038/s41467-020-18878-8

**Published:** 2020-10-09

**Authors:** Daniel M. Wilson, Yu Li, Amber LaPeruta, Michael Gamalinda, Ning Gao, John L. Woolford

**Affiliations:** 1 Department of Biological Sciences, Carnegie Mellon University, Pittsburgh, PA 15213 USA; 2 State Key Laboratory of Membrane Biology, School of Life Science, Tsinghua University, Beijing, China; 3 Peking University-Tsinghua University-National Institute of Biological Sciences Joint Graduate Program, Beijing, China; 4 State Key Laboratory of Membrane Biology, Peking-Tsinghua Center for Life Sciences, School of Life Sciences, Peking University, Beijing, China; 5 Present Address: Bayer AG, Strategy and Business Consulting, Building B151, 51368 Leverkusen, Germany

**Keywords:** Ribosomal proteins, Cryoelectron microscopy, Ribosome

## Abstract

The nascent polypeptide exit tunnel (NPET) is a major functional center of 60S ribosomal subunits. However, little is known about how the NPET is constructed during ribosome assembly. We utilized molecular genetics, biochemistry, and cryo-electron microscopy (cryo-EM) to investigate the functions of two NPET-associated proteins, ribosomal protein uL4 and assembly factor Nog1, in NPET assembly. Structures of mutant pre-ribosomes lacking the tunnel domain of uL4 reveal a misassembled NPET, including an aberrantly flexible ribosomal RNA helix 74, resulting in at least three different blocks in 60S assembly. Structures of pre-ribosomes lacking the C-terminal extension of Nog1 demonstrate that this extension scaffolds the tunnel domain of uL4 in the NPET to help maintain stability in the core of pre-60S subunits. Our data reveal that uL4 and Nog1 work together in the maturation of ribosomal RNA helix 74, which is required to ensure proper construction of the NPET and 60S ribosomal subunits.

## Abstract (web summary)

The nascent polypeptide exit tunnel (NPET) is a functional center of the large ribosomal subunit through which the nascent polypeptide chains travel from the peptidyltransferase center (PTC). Here the authors provide structural insight into NPET maturation and how it is linked to other aspects of ribosome biogenesis.

## Introduction

Translation of the genetic code and catalysis of peptide bond formation to synthesize proteins is carried out by ribosomes. Assembly of ribosomes requires the transcription, folding, modification, and processing of ribosomal RNA (rRNA), and stable association of ribosomal proteins (RPs) facilitated by *trans*-acting assembly factors (AFs). In eukaryotes, this process takes place in three separate subcellular compartments beginning in the nucleolus, continuing in the nucleoplasm, and concluding in the cytoplasm. To ensure their proper function, these complex ribonucleoprotein particles must undergo accurate assembly of their functional centers including the decoding center (DC) in the small subunit (40S) and the peptidyltransferase center (PTC) and nascent polypeptide exit tunnel (NPET) in the large subunit (60S)^1,2^.

The NPET is the 90 angstrom-long conduit through which all nascent polypeptide chains travel from the PTC to the exterior of the 60S subunit^3^. It has become increasingly clear that the NPET functions as a sensor of both specific sequences in nascent polypeptides and small molecule effectors, including antibiotics, to modulate the activity of the PTC and regulate the synthesis of specific subsets of proteins^4–6^. The physicochemical properties of the NPET have also been shown to actively affect protein folding^7^. Thus, we are particularly interested to learn how cells ensure a properly constructed NPET.

**Fig. 1:**
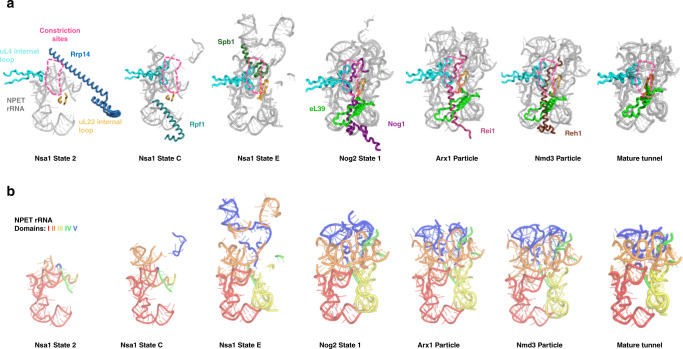
Ordered stages of NPET assembly. **a** Cryo-EM structures of preribosomes revealed that the NPET undergoes distinct stages of assembly. The Nsa1 state 2 particle (PDB: 6C0F) contains Rrp14 (sky blue) associated with an NPET precursor. The uL4 (cyan) and uL22 (bright orange) internal loops can be seen but the tunnel domain (TD) of uL4 is not yet visible. The Nsa1 state C particle (PDB: 6EM1) displays Rpf1 (teal green) in an incomplete NPET. The TD of uL4 (cyan) is now visible at this stage. The Nsa1 state E particle (PDB: 6ELZ) contains a more complete NPET. The CTD of Spb1 (dark green) can be seen toward the entrance of the NPET. In Nog2 particles (PDB: 3JCT), a nearly mature NPET, now containing eL39 (green), can be visualized with the CTD of Nog1 (dark purple) stretching almost the entire length of the NPET. The Arx1 (PDB: 5APN) and Nmd3 (PDB: 5H4P) particles reveal no further NPET rRNA maturation but show that the CTDs of Rei1 (dark pink) and Reh1 (brown), respectively, now occupy the NPET. **b** Five domains of rRNA secondary structure make up the NPET. In Nsa1 state C, the NPET contains mostly domains I (red) and II (orange). A small portion of domain IV (green) can also be seen and domain V (blue) has not yet folded into the NPET. In Nsa1 state E, domain III (yellow) has folded into a stable conformation to join the NPET. In Nog2 state 1, domain V has stably folded into the NPET while a portion of domain II has also folded into a near mature state. Minor rearrangements in NPET rRNA can be observed in Arx1, Nmd3, and mature particles (PDB: 4V88). For clarity, only NPET-relevant portions of proteins and rRNA are shown.

The crystal structure of mature yeast ribosomes first revealed that the eukaryotic NPET is composed of rRNA from five out of six domains of 25S rRNA, and the RPs uL4 (rpL4), uL22 (rpL17), and eL39 (rpL39)^8^. Some of the most conserved elements are the constriction sites formed by the internal loops of uL4 and uL22. In eukaryotes, the bilobal tunnel domain (TD) at the tip of the uL4 internal loop forms two constriction sites as opposed to one found in bacteria (Fig. 1a)^9^. The internal loops of both uL4 and uL22 have each been shown to be important for the assembly and function of ribosomes in both prokaryotes and eukaryotes^10–14^. Additionally, eL39 further distinguishes eukaryotic from prokaryotic NPETs and can help explain differences between translation modes observed between the two^9^.

Recent cryo-electron microscopy (cryo-EM) of native yeast pre-60S subunits has provided numerous structural snapshots of assembly intermediates, including glimpses of ordered stages of NPET construction (Fig. 1a, b). In early nucleolar pre-60S particles (Nsa1 state 2), a precursor to the tunnel is visible, with the long alpha helix of AF Rrp14 sterically hindering incorporation of 25S rRNA domain II into the NPET (Fig. 1a)^15^. At this stage, uL4 is present in preribosomes but the TD of uL4 is not visible and thus is likely to be in a flexible state. Next, in Nsa1 state C particles, Rrp14 has been released, the amino-terminal domain of AF Rpf1 contacts portions of 25S rRNA domain I that will become part of the tunnel, and the uL4 TD has stabilized and becomes visible. A more complete tunnel-like structure forms in later nucleolar intermediates. In Nsa1 state E particles, Rpf1 has been released and the CTD of AF Spb1 traverses the upper portion of the tunnel, contacting the TD of uL4 at the first constriction site, possibly functioning to further stabilize uL4 in the NPET^16^. As pre-60S particles transition from the nucleolus to the nucleoplasm, several AFs are released and others join pre-60S subunits. In the ensuing Nog2 state 1 particles, the CTD of AF Nog1 can be seen occupying almost the full length of the tunnel, contacting the uL4 TD^17^, and eL39 is recruited to the tunnel exit. Upon release of Spb1, insertion of the Nog1 CTD may serve to maintain stability of the uL4 TD. Once preribosomes are exported to the cytoplasm (Arx1 particles), Nog1 is released by the AAA-ATPase Drg1^18,19^ and replaced in the NPET by the CTD of AF Rei1. Rei1 is then removed and replaced in the NPET by its homologue Reh1 (Nmd3 particles), which is released upon completion of 60S subunit assembly^20–22^. The CTDs of both Rei1 and Reh1 also contact the TD of uL4. Thus, remarkably, the NPET is sequentially occupied by carboxy or amino-terminal extensions of AFs from early nucleolar stages of 60S subunit assembly until final steps in the cytoplasm.

Despite this wealth of structural information, it remains unclear precisely how the NPET is constructed, what are the specific roles of ribosomal proteins and assembly factors in NPET assembly, and how NPET formation fits into the hierarchy of 60S subunit biogenesis. To investigate these questions, we focused on uL4 and Nog1, which contact each other inside the NPET during late nucleolar and nucleoplasmic (middle) stages of assembly (Fig. 1).

We used biochemical and molecular genetic analyses, together with cryo-EM, to investigate the effects on ribosome assembly when we deleted the TD of uL4 or the Nog1 CTD. We found that pre-60S particles lacking the TD of uL4 harbor a misassembled NPET that lacked the densities of eL39 and the Nog1 CTD and are blocked or stalled in at least three different stages of 60S subunit assembly. These blocks begin with rRNA helix H74 (H74), which lies in close proximity to the TD of uL4, being improperly assembled. Consequently, adjacent helices H75, H76, and H68, are also displaced. We also found that the Nog1 CTD functions to stabilize the uL4 TD and ensure proper maturation of H74. Thus, truncation of the Nog1 CTD causes an assembly defect similar, but not identical to that of deleting the uL4 TD. Our work suggests that the quality of the NPET is monitored throughout assembly of the 60S subunit, and errors in its construction can be propagated through rRNA.

## Results

### The uL4 tunnel domain is necessary for 60S assembly

**Fig. 2:**
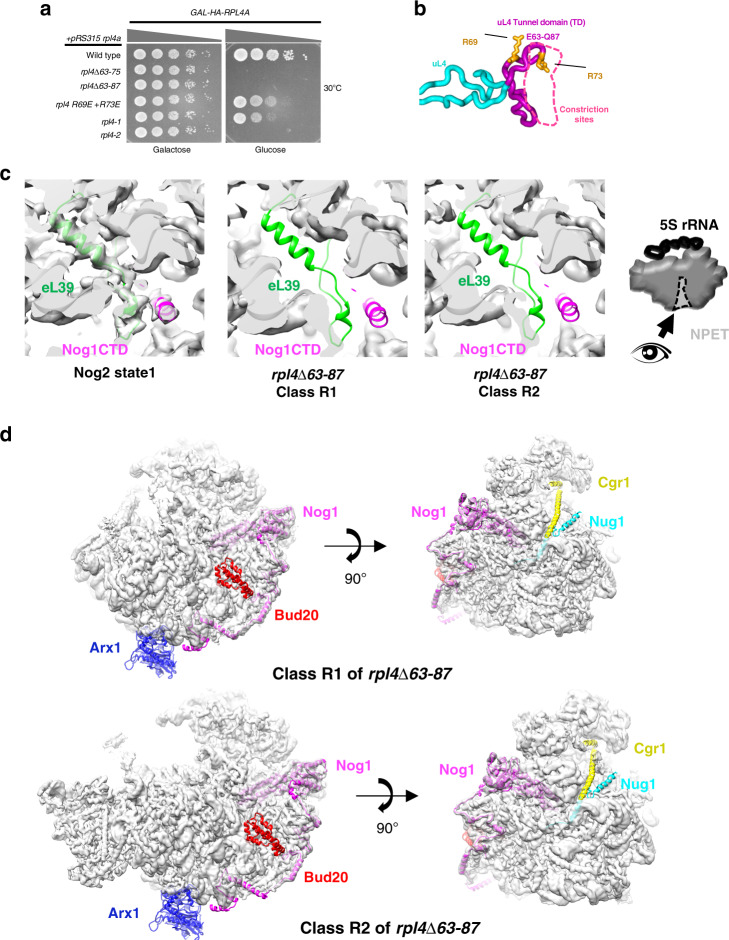
Cryo-EM of *rpl4*∆*63–87* Nog2-associated mutant particles. **a** Deletion of the uL4 tunnel domain (aa 63–87) is lethal. Each strain contains *GAL-RPL4* that can be turned off by shifting cells from galactose to glucose-medium, and a plasmid that is constitutively expressing an *rpl4* mutant allele. The *rpl4-1* and *rpl4-2* mutantions are alanine scans of residues 63–68 and 69–74, respectively. Serial dilutions (1:10 to 1:10,000) of cultures were pipetted onto selective medium containing either galactose or glucose, incubated at 30 °C, and imaged after 3 days. **b** Diagram of the internal loop of uL4 (cyan), including the TD (purple) that extends into the NPET to help create the constriction sites (dotted pink line). Indicated arginine residues (orange) were mutated to glutamate. The TD forms the constriction sites in the NPET (pink dotted line). **c** Close up view of the exit of the NPET in wild-type Nog2 state 1 and *rpl4∆63–87* classes R1 and R2. Gray densities are not present in classes R1 and R2 in the spaces where eL39 and the Nog1 CTD should be. Thus, the exposed cartoon model indicates missing components. **d** View of *rpl4∆63–87* classes R1 and R2 from the subunit interface (left) and solvent side (right). Densities are fitted to the atomic model of wild-type Nog2 state 1 (PDB: 3jct). Exposed cartoon models of Arx1 (blue), the Nog1 CTD (magenta), Bud20 (red), Cgr1 (yellow), and Nug1 (cyan) represent missing densities for each respective protein.

Previously, the tunnel domain (TD) of uL4 was shown to be necessary for 60S subunit assembly^12^. However, its precise function during ribosome biogenesis remains unclear. Because the bilobal TD of uL4 (amino acids 63–87) forms constriction sites in the NPET and has more recently has been shown to interact with a number of tunnel-probing ribosome assembly factors^15–17,21,22^, we wanted to investigate its specific role in 60S subunit assembly in greater detail. To this end, we have characterized multiple strains expressing different mutations or full deletions of the uL4 TD. We focused on the effects of the *rpl4*∆*63–87* mutation because it removes both constriction sites from the NPET (Fig. 2a, b). To assay effects of the *rpl4*∆*63–87* mutation, we expressed the mutant uL4 protein from a plasmid in a strain conditional for expression of endogenous wild-type uL4 (*GAL-RPL4*). This strain grows at wild-type rates on galactose-medium but fails to grow on glucose-medium where only the mutant protein is expressed.

In order to determine which stages of the 60S subunit assembly pathway are affected in the *rpl4*∆*63–87* mutant, we assayed pre-rRNA processing using primer extension and northern blotting. Compared to the wild-type strain, the *rpl4*∆*63–87* mutant accumulated both 27SB and 7S pre-rRNAs, which normally undergo processing during late nucleolar and nucleoplasmic stages of 60S assembly (Supplementary Fig. 1a). To confirm that these middle steps of 60S subunit assembly are affected in the *rpl4*∆*63–87* mutant, we assayed localization of pre-60S subunits using uL23-eGFP (rpL25), a reporter of both mature and pre-60S subunits, and Nop1-mRFP, a nucleolar marker. Relative to the wild-type strain, we observed accumulation of pre-60S subunits in the nucleolus and nucleoplasm in the *rpl4*∆*63–87* mutant (Supplementary Fig. 1b). These results demonstrate that the uL4 TD is necessary for late nucleolar and nucleoplasmic stages of 60S subunit assembly prior to pre-60S export from the nucleoplasm.

### The tunnel domain of uL4 stabilizes rRNA

Because our assays thus far suggest a defect during late nucleolar/nucleoplasmic stages of 60S assembly, we used the late nucleolar/nucleoplasmic acting AF Nog2 as bait to affinity-purify particles from this stage of assembly. We performed cryo-EM on these Nog2-associated pre-60S particles, which enabled observation of both small and dramatic changes in rRNA and protein conformation that would otherwise be undetectable by other methods.

Previously, cryo-EM structures of wild-type Nog2-associated particles revealed three distinct consecutive assembly intermediates during middle stages of 60S subunit assembly: Nog2 states 1, 2, and 3. Nog2 state 1 particles contain a prerotated 5S RNP, Nog2 state 2 contains a rotated 5S RNP along with AFs Sda1, the Rix1 complex, and Rea1^23^, while Nog2 state 3 contains a rotated, near mature 5S RNP and has released Sda1, the Rix1 subcomplex, Rea1, and Rsa4. Therefore, using Nog2 as bait enabled us to assess the progress of 60S subunit assembly during late nucleolar and nucleoplasmic stages.

Through cryo-EM 3D classification, we obtained seven states for the *rpl4*∆*63–87* mutant particles, which we refer to as classes R1-R7 (Supplementary Fig. 2a, b) As expected, density for the deleted TD of uL4 could not be observed in any of these classes. Classes R3 and R4 comprise a small fraction of the particles (10%), and represent intermediates inhibited during early steps of 60S subunit maturation (Supplementary Fig. 3a, b) (see Discussion). The R1 and R2 particles (27% and 12%, respectively) closely resemble Nog2 state 1 particles, containing a prerotated 5S RNP, and thus have progressed beyond the stages represented by the R3 and R4 particles. The R7 class (10%) is R1-like but lacks densities for nearly the entire domains III and IV of 25S rRNA and appears to represent R1 particles undergoing turnover (Supplementary Fig. 3e). Class R6 particles contain a rotated 5S RNP and resemble Nog2 state 3 (Arx1 particles) (Supplementary Fig. 3d), but also lack densities for several rRNA helices underneath the L1 stalk. Thus, R6 particles appear to be blocked later than class R1, after rotation of the 5S RNP. Finally, class R5 resembles R6 but, lacks densities for nearly the entire domains III, IV, and V of 25S rRNA and therefore may represent R6 particles undergoing turnover (Supplementary Fig. 3c).

We first focused on the preribosomes in classes R1 and R2 because they are the most abundant and stable mutant particles. The structures of these R1 and R2 particles were solved at a resolution of 3.2 and 3.3 Å (Supplementary Fig. 2c, d). In classes R1 and R2, no density could be observed for the NPET-occupying RP eL39, the Nog1 CTD, or AF Arx1 that binds to the NPET exit platform (Fig. 2c, d). This indicates that lack of the L4 TD causes significant disruption in the composition of the immature NPET.

Several other protein components were also found to be missing or flexible in both classes R1 and R2 (Fig. 2d). We could not observe densities for Bud20, Nug1, and Cgr1. While Cgr1 is known to be necessary for 5S RNP rotation^24^, it is not always detected in relevant pre-60S particles, even in wild-type Nog2 particles^17^. This suggests that Cgr1 may be loosely bound to pre-60S subunits and may explain why it cannot be observed in *rpl4*∆*63–87* mutant particles.

**Fig. 3:**
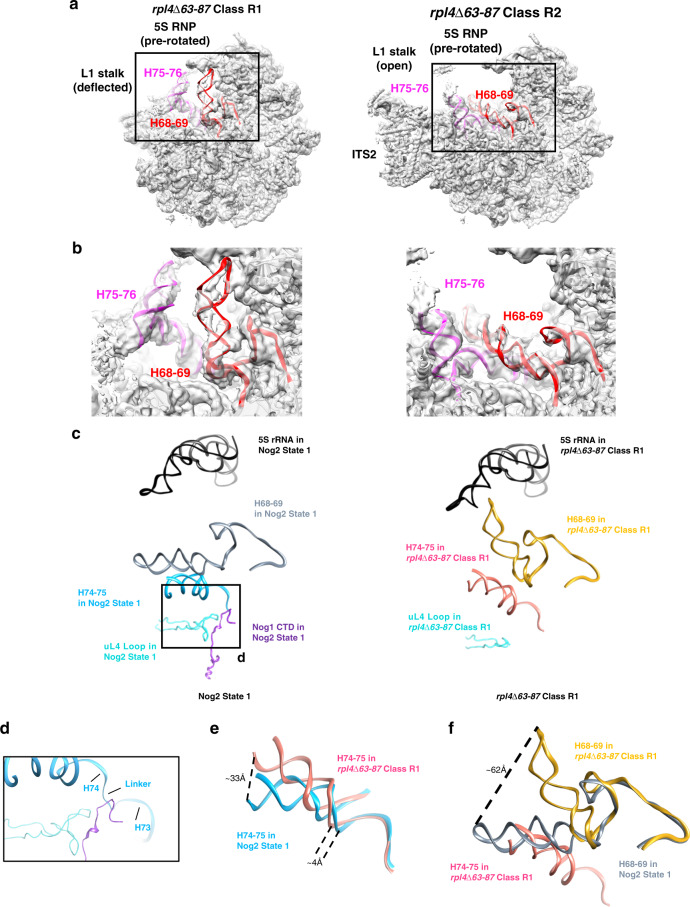
Aberrant conformations of rRNA helices in *rpl4*∆*63–87* mutant particles. **a** Two major density maps (R1 and R2 particles, shown in light gray) obtained from Nog2-associated *rpl4*∆*63–87* particles are overlaid with their respective atomic models. **b** Enlarged view of the conformational changes in of rRNA helices from the two major classes of the *rpl4*∆*63–87* mutant. Compared with wild-type Nog2-particles (state 1), H75-76 (magenta) and H68-69 (red) have undergone dramatic conformational changes in class R1 but not in class R2. **c** rRNA helices from the atomic model of wild-type Nog2 state 1 particles are compared with those in the atomic model of the *rpl4*∆*63–87* mutant class R1. Positions of relevant rRNA helices in relation to uL4 and Nog1 in the NPET in *RPL4* wild-type (left) and *rpl4*∆*63–87* mutant (right) Nog2 particles. Helix 74–75 (light blue for wild-type and hot pink for mutant) extend into the NPET. H74 is located in close proximity to the uL4 TD (cyan) and the CTD of Nog1 (dark purple). H74-75 are located below H68-69 (gray for wild-type and gold for mutant). The 5S rRNA (black) is shown to provide a frame of reference. **d** Inset showing the interaction of the TD of uL4, the Nog1 CTD, and the rRNA linker between H73 and H74. **e** H74-75 from *rpl4∆63–87* mutant particles (hot pink) are shifted relative to those in wild-type Nog2 state 1 particles (light blue). H74 is displaced up to ~4 Å while H75 is shifted up to ~33 Å. **f** H75 in *rpl4*∆*63–87* mutant particles is shifted to an aberrant position (hot pink) that clashes with the native position of H68 (gray). This displaces H68 in *rpl4*∆*63–87* mutant particles (gold), causing it to shift up to ~62 Å.

We next looked at effects of the *rpl4*∆*63–87* mutation on the structure of pre-rRNA in Nog2 particles. Although class R2 exhibits wild-type rRNA conformations, significant shifts in rRNA helices 68–69 and 74–79 could be seen in class R1 (Fig. 3a, b). Further analysis of the atomic model of class R1 revealed how a misassembled NPET could cause these rRNA conformational changes (Fig. 3c). These aberrant shifts begin with a loss of the interaction between the TD of uL4, the Nog1 CTD, and the linker between rRNA helices 73 and 74 (Fig. 3d). Recent cryo-EM structures further suggest the importance of this interaction^25^. H74 is displaced ~4 Å relative to the wild-type conformation, indicating that this portion the NPET is improperly assembled (Fig. 3e). The conformational change of H74 affects the adjacent H75, which can be seen shifted up to ~33 Å from its native position in Nog2 state 1 (Fig. 3e). In this aberrant position, H75 clashes with H68 and displaces it up to ~62 Å toward the 5S rRNA (Fig. 3f). H68 is the binding site for AF Sda1, which is important for rotation of the 5S RNP in Nog2 particles and acts as an intersubunit bridge in mature wild-type ribosomes. Once structured in Nog2 state 1, this helix remains stably docked throughout middle and later stages of 60S subunit assembly^8,17,21,22,26^. Therefore, deviations from this stable position of H68, as seen in class R1, likely have negative consequences as a result of improper NPET construction during the assembly of 60S subunits.

Consistent with the displaced H74-75, the 3-way junction of rRNA helices 75, 76, and 79 is affected in class R1. Specifically, the L1 stalk (H76 and uL1) adopts an unusual, deflected conformation not observed in any previously characterized wild-type particles (Supplementary Fig. 4) and H79 is unstructured. As a result, the internal loop of uL15, which interacts with H75 in Nog2 state 1, cannot be visualized. Likewise, the N-terminus of eL8, which interacts with the 3-way junction of H75, H76, and H79 and was previously shown to be necessary for middle stages of 60S subunit assembly^27^, is also flexible (Supplementary Fig. 5a–d). These observations further characterize the perturbance of this 3-way junction as a result of improper NPET construction.

### A misassembled NPET can block Sda1 binding to the pre-60S

**Fig. 4:**
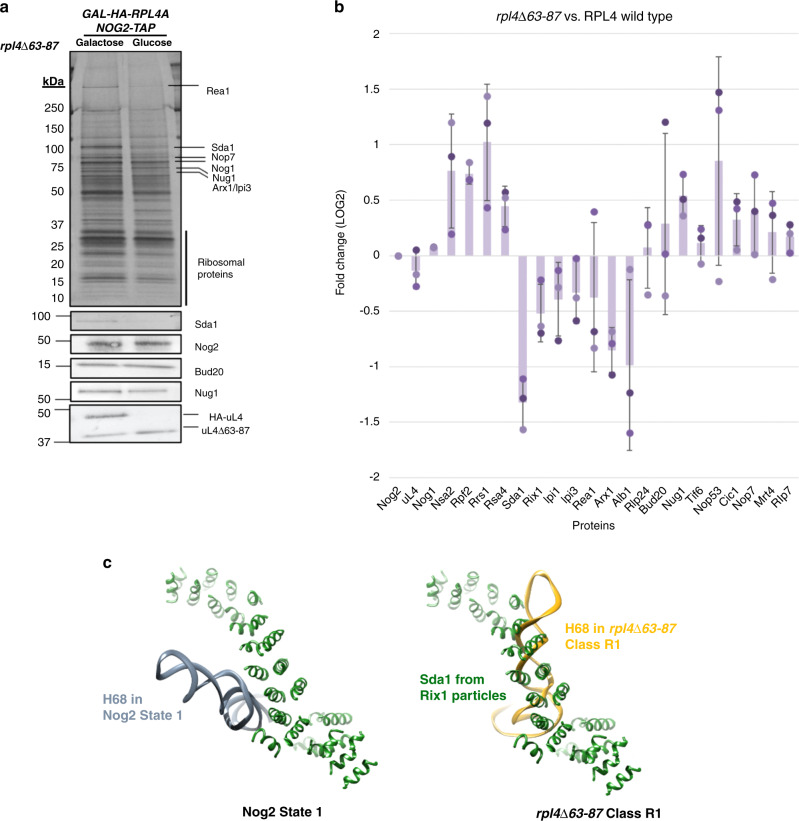
Deviations in rRNA structure in *rpl4*∆*63–87* mutant particles result in H68 clashing with the binding site of Sda1. **a** SDS-PAGE gels followed by silver staining or western blotting show proteins in Nog2-associated particles purified from mutant *rpl4*∆*63–87* cells. Labeled bands were identified using mass spectrometry. **b** Samples prepared as in panel a were scaled up and subjected to semi-quantitative iTRAQ mass spectrometry to assay changes in relative amounts of pre-60S subunit proteins. Proteins were labeled with iTRAQ reagents and compared to wild-type counterparts. Each bar represents a biological replicate. All protein levels are normalized to the bait protein, Nog2. Ratios are represented on a log_2_ scale. Error bars represent standard deviation (*n* = 3). **c** The AF Sda1 (dark green) from Rix1 particles superimposed over wild-type Nog2 state 1 particles (left) and *rpl4*∆*63–87* mutant particles (right). H68 (gray for wild-type and gold for mutant) in *rpl4*∆*63–87* mutant particles clashes with almost the full length of observable Sda1.

To help clarify the consequences of the aberrant rRNA conformations observed in *rpl4*∆*63–87* mutant pre-60S subunits, we utilized SDS-PAGE, western blotting, and iTRAQ mass spectrometry to analyze the protein composition of these particles. Importantly, levels of uL4 do not change in the *rpl4*∆*63–87* mutant preribosomes compared to wild-type, indicating that the mutant uL4 protein is stable and able to efficiently assemble into pre-60S subunits. However, we did observe a number of changes in amounts of other proteins in the mutant preribosomes. Most striking was that levels of Sda1 decreased and levels of Rpf2 and Rrs1 increased (Fig. 4a, b). In wild-type cells, the AFs Rpf2 and Rrs1, which are bound to the 5S RNP, are thought to exit from Nog2 particles before Sda1 binds to pre-60S subunits^28^. Thus, Sda1 fails to associate with a significant fraction of pre-60S subunits in this mutant, and Rpf2 and Rrs1 fail to exit from preribosomes. Furthermore, amounts of downstream AFs Rea1, and the Rix1 complex (Rix1, Ipi1, and Ipi3), which depend on Sda1 for recruitment onto pre-60S subunits and are required to stabilize 5S RNP in its rotated state, also are decreased relative to wild-type (Fig. 4b). Consistent with a failure of Rea1 to enter assembling pre-60S subunits, levels of Rsa4, which is removed by Rea1, are increased.

### The C-terminal domain of Nog1 facilitates 60S assembly

**Fig. 5:**
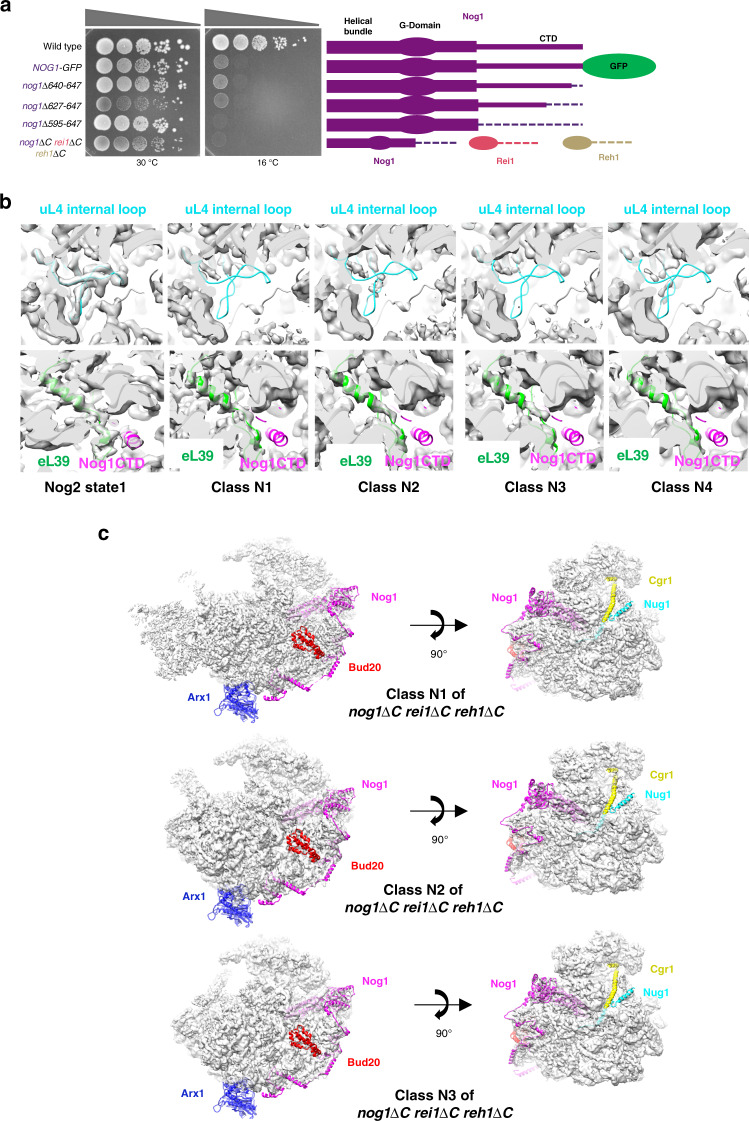
The Nog1 CTD stabilizes the tunnel domain of uL4. **a** Serial dilutions (1:10 to 1:10,000) of *NOG1* wild-type and *nog1* mutant cultures were spotted onto solid medium containing glucose, and incubated at 30 °C or 16 °C, for 8 days. Each construct is depicted in cartoon form (right). **b** Density maps of the wild-type Nog2 state 1 and N1–N4 mutant particles are aligned with the atomic model of wild-type Nog2 state 1 particles, displaying the interior of the NPET. Densities (gray) for the uL4 TD (cyan) are missing in the NPET in all four mutant classes (top). Densities for eL39 (green) can be visualized in classes N1–N4 but no densities for the Nog1 CTD (magenta) are visible (bottom). **c** View of *nog1*∆*C rei1*∆*C reh1*∆*C* classes N1–N3 from the subunit interface. Densities are fitted to the atomic model of wild-type Nog2 state 1 (PDB: 3jct). Exposed cartoon models of Arx1 (blue), the Nog1 CTD (magenta), Bud20 (red), Cgr1 (yellow), and Nug1 (cyan) represent missing densities for each respective protein.

Since the Nog1 CTD is in close contact with the TD of uL4 in wild-type cells and fails to enter the NPET in *rpl4*∆*63–87* mutant particles, we wanted to investigate whether deleting the Nog1 CTD results in similar or different 60S subunit assembly defects compared to those observed in the *rpl4*∆*63–87* mutant. Thus, we generated a series of genomic mutations to sequentially truncate the CTD of Nog1 (Fig. 5a and Supplementary Fig. 6a). We also constructed the triple mutant *nog1*Δ*595–647 rei1*Δ*341-393 reh1*Δ *380-432 (nog1*Δ*C rei1*Δ*C reh1*Δ*C)* lacking the NPET-occupying sequences of Nog1, Rei1, and Reh1. As an orthogonal approach to deleting Nog1 sequences inserted into the tunnel, we constructed a strain expressing Nog1 fused to GFP at its C-terminus (*NOG1-GFP*). Because an immature NPET is already formed before the Nog1 CTD enters the tunnel (Fig. 1a), this bulky GFP tag must prevent insertion of the Nog1 CTD into the tunnel.

All of these mutants and the *NOG1-GFP* strain exhibit an extreme cold-sensitive growth defect (Fig. 5a). In contrast, an analogous C-terminal truncation of Rei1 has been shown to have minimal effects on cell growth at all temperatures, while the complete absence of Reh1 has no obvious growth defect^22,29^. To examine whether this cold-sensitivity reflects a defect in 60S subunit assembly, we used sucrose gradient fractionation to assay amounts of free ribosomal subunits, 80S monosomes, and polyribosomes in extracts prepared from each strain. In each case, levels of free 60S ribosomal subunits are decreased relative to 40S subunits, polyribosomes are decreased, and halfmer polyribosomes are present, indicating a defect in production of 60S subunits (Supplementary Fig. 6b).

To determine which interval of 60S subunit assembly is perturbed in each mutant, we examined pre-rRNA processing. When cells were shifted to 16 °C for 5 h, 27SB pre-rRNA accumulates in all mutant strains relative to the wild-type *NOG1* yeast strain (Supplementary Fig. 6c). Together, these results indicate that the presence of the CTD of Nog1 in the NPET is important during late nucleolar and nucleoplasmic stages of 60S subunit assembly.

### The C-terminal domain of Nog1 scaffolds uL4

**Fig. 6:**
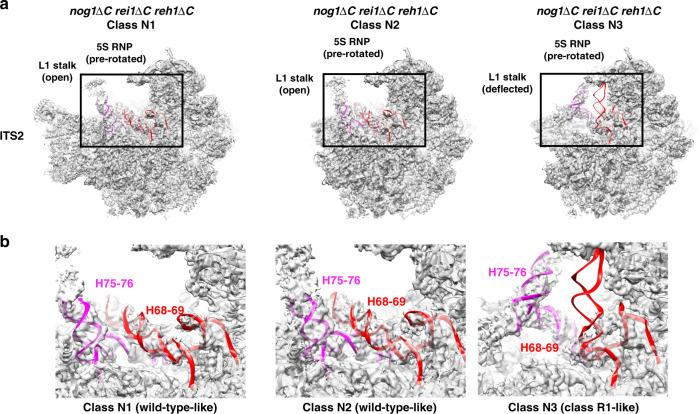
A minority of *nog1*∆*C rei1*∆*C reh1*∆*C* mutant pre-60S particles display aberrant rRNA conformations. **a** Densities of classes N1–N3 of *nog1*∆*C rei1*∆*C reh1*∆*C* mutant particles solved at a resolution of 3.0–6.0 Å. Classes N1 and N2 are aligned with the atomic model of class R2, while class N3 is aligned with the atomic model of class R1. Note that ITS2 is missing from classes N2 and N3. **b** View of H68-69 (red) and H75-76 (magenta) from classes N1–N3.

**Fig. 7:**
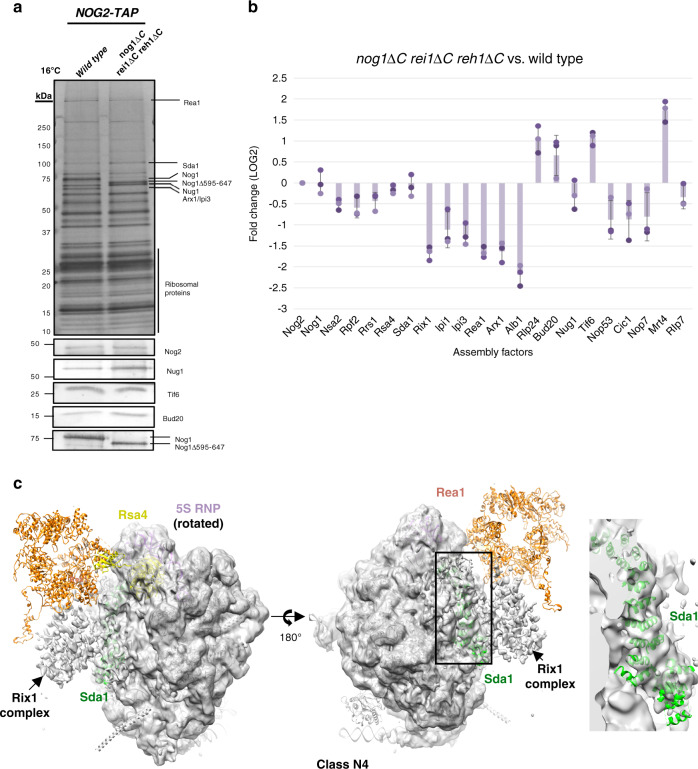
Truncation of the Nog1 CTD may affect closing of the L1 stalk. **a** SDS-PAGE of proteins in Nog2-associated preribosomes affinity purified from wild-type and *nog1*∆*C rei1*∆*C reh1*∆*C* mutants shifted to 16 °C for 5 h. Proteins in labeled bands were identified by mass spectrometry. **b** Semi-quantitative iTRAQ mass spectrometry reveals differences in relative amounts of proteins labeled with iTRAQ reagents in Nog2-particles affinity purified from the *nog1*∆*C rei1*∆*C reh1*∆*C* mutant compared to those in wild-type particles. Each bar represents a biological replicate. All protein levels are normalized to the bait protein, Nog2. Ratios are represented on a log_2_ scale. Error bars represent standard deviation (*n* = 3). **c** Densities for class N4 are fitted to the atomic model of the Rix1 particle (PDB: 5fl8). Density for Sda1 (green) can be seen in the particles. The 5S RNP (purple) is in the rotated state in class N4. Weak density can be seen for the Rix1 complex, and Rea1 (orange) is almost completely missing. Rsa4 (yellow) is visible in the particles.

We performed cryo-EM of Nog2-containing particles affinity purified from the *nog1*∆*C rei1*∆*C reh1*∆*C* mutant, shifted to 16 °C for 5 h. We focused on this triple mutant because it exhibited the most severe growth defect and we expected that the CTD’s of Rei1 and Reh1 might partially rescue defects of a *nog1*∆*595–647* mutant. We obtained structures of four stable intermediates at resolutions ranging from 3.0 to 4.3 Å, which we refer to as classes N1–N4 (Supplementary Figs. 7 and 8). The most striking observation from these structures is that the interior of the NPET in all four *nog1*∆*C rei1*∆*C reh1*∆*C* particle classes lacks density for the TD of uL4 (Fig. 5b). This result indicates that the presence of the Nog1 CTD in the NPET is required to stabilize the TD of uL4. In contrast to the *rpl4*∆*63–87* mutant particles, eL39 was present in classes N1–N4 (Fig. 5b). Classes N1 and N2 closely resemble class R2 from the *rpl4*∆*63–87* mutant, except that the ITS2 structure is absent in class N2 (Fig. 5c). This latter observation is consistent with previous findings that removal of ITS2 can occur independently from other remodeling events such as 5S RNP rotation^30,31^. Class N3 particles resemble class R1 from the *rpl4*∆*63–87* mutant, including the aberrant rRNA rearrangements, deflected L1 stalk, and absence of Sda1 (Fig. 6a, b). However, while class R1 makes up about 27% of total particles obtained from the *rpl4*∆*63–87* mutant, class N3 only comprises about 7% of total particles recovered from the *nog1*∆*C rei1*∆*C reh1*∆*C* mutant. Most notable is class N4. Unlike any classes observed in the *rpl4∆63–87* mutant, class N4 represents improperly assembled Nog2 state 2 particles; it displays weak density for the Rix1 complex and lacks density for Rea1. Consistent with inefficient recruitment of the Rix1 complex and Rea1, the Rea1-dependent removal of Rsa4 has not yet occurred in class N4 (Fig. 7c). These results indicate that the *nog1*∆*C* *rei1*∆*C reh1*∆*C* mutant is distinct from the *rpl4*∆*63–87* mutant. In this mutant, a smaller fraction of intermediates appears to be blocked at the stage of Nog2 state 1, and the second block appears to be at the state 2 stage, rather than the state 3 stage, of Nog2 particles. These differences between the *rpl4*∆*63–87* mutant and the *nog1*∆*C* *rei1*∆*C reh1*∆*C* mutant may in part result from the absence of the TD of uL4 in the former, versus its being present but in a flexible conformation in the latter.

To further characterize the differences between *rpl4*∆*63–87* and *nog1*∆*C rei1*∆*C reh1*∆*C* mutant particles, we assessed the protein composition of Nog2-associated pre-60S subunits using SDS-PAGE, western blotting, and iTRAQ mass spectrometry. Purifications were done using wild-type or mutant cells grown at 30 °C and then shifted to 16 °C for five hours. SDS-PAGE followed by silver staining or western blotting indicated that deletion of the Nog1 CTD does not affect the stability or recruitment of Nog1 into pre-60S subunits (Fig. 7a). In contrast to the *rpl4*∆*63–87* mutant, levels of Sda1, Rpf2, and Rrs1 do not change in *nog1*Δ*C rei1*Δ*C reh1*Δ*C* mutant particles relative to wild-type cells (Fig. 7b). Despite the observation that both the exit of Rpf2 and Rrs1 and the entry of Sda1 are unaffected in the *nog1*Δ*C rei1*Δ*C reh1*Δ*C* mutant, levels of the Rix1 complex and Rea1, which are required for stabilization of the 5S RNP in its rotated state, are consistently decreased relative to wild-type (Fig. 7b). These results indicate that, for reasons that are still not entirely clear, *nog1*Δ*C rei1*Δ*C reh1*Δ*C* mutant pre-60S subunits are able to recruit Sda1 but cannot recruit the other machinery necessary for 5S RNP rotation as efficiently as wild-type particles. This unusual protein composition most closely reflects what was observed by cryo-EM of class N4 of the *nog1*∆*C re1*∆*C reh1*∆*C* mutant particles (Fig. 7c).

## Discussion

Here, we combined molecular genetics and biochemical approaches with high-resolution cryo-EM to uncover how construction of the NPET fits into the hierarchy of 60S ribosomal subunit assembly in yeast. Our work reveals that a misassembled NPET can delay or block at least three different stages of pre-60S subunit assembly and demonstrates interdependence between the TD of uL4 and the CTD of Nog1.

**Fig. 8:**
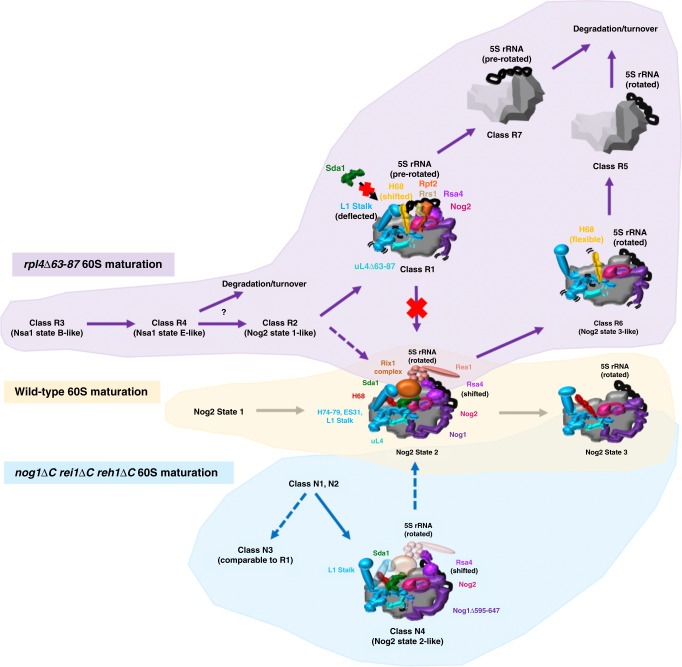
NPET maturation in the 60S subunit assembly hierarchy. In the wild-type 60S subunit assembly pathway (middle, tan), Nog2 state 1 progresses to Nog2 state 2 particles, which have bound AFs Sda1 (dark green), the Rix1 complex (orange), and Rea1 (coral). The L1 stalk has closed, and the 5S rRNA has rotated ~180°. Rsa4 (light purple) has also shifted to face Rea1. These wild-type particles progress to Nog2 state three particles, which have released multiple AFs and are ready to exchange Nog2 for Nmd3 and be exported from the nucleoplasm to the cytoplasm. In the *rpl4*∆63–87 mutant (top, purple) classes R3 and R4 are the earliest chronological particles to be observed. Some fraction presumably progresses to class R2. Class R2 particles are wild-type-like and likely progress to class R1 particles, which display a shifted rRNA helix H68 (gold) that prevents binding of Sda1 and progression to Nog2 state 2. These class R2 particles are likely precursors to class R7 on a pathway to turnover. Alternatively, some fraction of class R2 particles may progress past Nog2 state 2 to class R6, which displays aberrant conformations of multiple rRNA helices. Consequently, class R6 particles are likely precursors to class R5 on a pathway to turnover. In the *nog1*∆*C rei1*∆*C reh1*∆*C* mutant, wild-type-like classes N1 and N2 (bottom, teal) progress to either N3, which resembles class R1, or class N4. Class N4 primarily displays density for the L1 stalk in an open position and Sda1 is bound, but weak densities (transparent colors) can be seen for the Rix1 complex, Rea1, and the L1 stalk in a closed position. Some fraction of class N4 particles likely progresses to Nog2 state 3. Two curved black lines represent flexibility in protein or rRNA structure. Solid vs. dotted arrows represent major and minor pathways, respectively.

Previous work purifying Nog2-associated pre-60S particles from wild-type cells yielded three major stable assembly intermediates (Nog2 states 1–3)^17^, with 5S RNP rotation occurring in Nog2 state 2. Our cryo-EM analysis of Nog2-associated pre-60S particles from the *rpl4*∆*63–87* mutant revealed seven different particle states. Based on our data as well as others’ data, we propose a model to explain the chronological progression and fate of each individual particle class through the 60S subunit assembly pathway (Fig. 8).

Using the atomic model of class R1 from the *rpl4*∆*63–87* mutant, we were able to visualize several rRNA conformational changes that are apparently initiated by the instability of the linker between H73 and H74, which results in increased flexibility of H74 (Fig. 3c, 3d). In wild-type particles, H74 is one of the last rRNA helices in the NPET to undergo maturation (Fig. 1b). The absence of the uL4 TD results in a flexible H74, which appears to be sufficient to cause H75 to adopt an aberrant conformation. Consequently, H68 cannot remain stably docked on pre-60S subunits, and the 3-way junction of H75, H76, and H79 becomes destabilized, causing the L1 stalk to adopt a deflected position. Notably, densities for a shifted H75 could also be observed in classes R4 and R6 (Supplementary Fig. 9a). Consistent with this observation, densities for H68 are not visible in either class (Supplementary Fig. 9b), and the L1 stalk is deflected in class R4, the same as R1, indicating that L1 stalk maturation may be affected in early stages of 60S assembly. In summary, we suggest that a flexible H74, which occurs as a consequence of an improperly formed NPET, can shift rRNA helices 75, 76, and 68 toward aberrant conformations that can affect several stages of 60S subunit assembly.

Classes R3 and R4 resemble the early nucleolar intermediates Nsa1 state B and E, respectively, which and do not usually co-purify with Nog2 particles^16,17^. However, class R4 contains density for the bait protein Nog2 and is therefore unlikely to be a contaminant. Nog2 may be entering pre-60S particles earlier due to their stalling^32^. This defect in early particles resulting from the absence of the TD of uL4 is consistent with the observation that the TD of uL4 is first visible (and therefore present in a stable form) in the NPET at this stage of wild-type 60S subunit maturation (Fig. 1a). Class R4 lacks densities for several AFs typically found in State E particles, including Noc3, Spb1, Brx1, and Ebp2. These AFs have most likely already associated and dissociated from pre-60S particles, while the AFs still remaining on particles (Erb1, Ytm1, and Has1) have not yet been released. Otherwise, failure of so many essential AFs to initially bind early assembly intermediates would result in their rapid turnover^33^. It remains unclear what the biological consequences of a deflected L1 stalk in class R4 may be. Therefore, classes R3 and R4 may represent on-pathway particles that are delayed in their progression to middle stages of assembly.

The *rpl4*∆*63–87* mutant particles that reach class R2 may subsequently become trapped in class R1, be unable to bind Sda1, and become destined for turnover (class R7). Alternatively, they might progress beyond the 5 S RNP rotation checkpoint to class R6. Because densities for H68 are not visible in class R6, we suspect that this flexible intersubunit bridge results in particles destined for turnover (class R5). However, in the case of R6, this flexibility of H68 may not arise until after 5S RNP rotation occurs (Fig. 8). Together, our data suggest that proper maturation of the NPET affects the stability of specific rRNA helices and keeps pre-60S particles on-pathway. These mutant classes blocked at various stages may reflect the phenomenon of rerouting ribosome assembly to alternate pathways in response to stress^34,35^.

Deletion of the TD of uL4 consistently resulted in more extreme phenotypes than those observed in *nog1* CTD mutants. For example, the *rpl4*∆*63–87* mutant is lethal at all temperatures, whereas the nog1 CTD mutants exhibited severely reduced growth only at 16 °C (Figs. 2a, 5a). The different extent to which maturation of pre-60S subunits is blocked in *rpl4* compared to *nog1* mutants may reflect the difference between the absence of the uL4 TD (*rpl4*∆*63–87*) vs. the TD being present but in a flexible state (*nog1*Δ*C rei1*Δ*C reh1*Δ*C*). This milder effect in the *nog1* mutant is also consistent with the observation that class N3, which contains the same aberrant rRNA conformations as class R1, makes up only 7% of the total particles obtained from the *nog1*∆*C rei1*∆*C reh1*∆*C* while class R1 makes up 27% of *rpl4*∆*63–87* mutant particles (Supplementary Figs. 2b and 7b). When flexible, the TD of uL4 might still stabilize H74 enough to maintain growth at 30 °C in rich media. However, the potential for this rRNA to become trapped in unproductive rRNA conformations might increase at lower temperatures and result in cold sensitivity^36^. Alternatively, or in addition, the lack of eL39 in the NPET of *rpl4*∆*63–87* mutant particles could help explain the difference between the *rpl4* and *nog1* mutants. While eL39 is not essential for growth, *rpl39*∆ mutants do exhibit growth defects^37^. Although it is unclear how eL39 is loaded into pre-60S subunits and why exactly deletion of the uL4 TD prevents this, eL39 may fine-tune NPET assembly.

Our results also suggest that Nog1 plays the most important role of the three tunnel-occupying AFs Nog1, Rei1, and Reh1 during ribosome assembly. Analogous C-terminal truncation of Rei1 has minimal effects on cell growth at all temperatures, while *reh1∆* cells have no obvious growth defect^22,29^. Nevertheless, our triple truncation mutant, *nog1*Δ*C rei1*Δ*C reh1*Δ*C*, displayed a slightly slower growth rate at 30 °C than the *nog1*∆*595–647* single mutant. This leaves the possibility that the NPET-occupying amino acids in the CTDs of either Rei1 or Reh1 perform a still unknown function.

Class N4 particles from the *nog1*∆*C rei1*∆*C reh1*∆*C* mutant resemble Nog2 state 2 particles but lack density for Rea1 and display weak density for the Rix1 complex (Fig. 7c). The only other deviation from wild-type Nog2 state 2 in class N4 is that the L1 stalk adopts an open conformation as opposed to a closed one. While this particle makes up a minor fraction of total particles (5.6%), it is possible that failure to close the L1 stalk may explain why the Rix1 complex and Rea1 are not stably associated with pre-60S subunits. In this scenario, in wild-type cells, both Sda1 binding and closure of the L1 stalk may promote recruitment of the Rix1 complex and Rea1. The flexible uL4 TD in the *nog1*∆*C rei1*∆*C reh1*∆*C* mutant may subtly perturb the conformation of H74, which could be propagated to H75 and then to H76 in the L1 stalk. These perturbations might affect the closing of the L1 stalk in ways that we do not yet fully understand.

A consistent defect observed across all cryo-EM classes from both mutants was the lack of the nonessential AF and export adaptor, Arx1 (Figs. 2d and 5c). Portions of the Nog1 CTD that lie just outside the NPET interact with Arx1^17^. We reasoned that the flexibility or absence of the Nog1 CTD in our mutants may prevent stable assembly of Arx1. Normally, Rei1 is necessary for the removal of Arx1 from pre-60S subunits. When this fails to occur, Arx1 remains bound to preribosomes as they are exported to the cytoplasm. However, when Arx1 cannot assemble into preribosomes, it is found in the nucleoplasm^38^. Consistent with the assembly defect of Arx1, we observed that upon truncation or tagging of the Nog1 CTD in an *rei1*∆ strain, Arx1-GFP was located in the nucleoplasm (Supplementary Fig. 10a, b). These results indicate that the Nog1 CTD recruits Arx1 to pre-60S subunits, contributing to the export competency of pre-60S subunits.

It is vital to the cell’s survival that the constriction sites in the NPET are assembled properly, since these sites impact protein synthesis. In bacteria, deletion of the internal loop of uL4 results in a cold-sensitive growth defect and a failure to respond to the *cmlA*^*Crb*^ pausing peptide during protein synthesis^10^. Mutations in the NPET have also been shown to confer resistance to the macrolide antibiotic, erythromycin^14,39^. Several previous results offered early clues that mechanisms may operate to couple NPET construction with other remodeling events during 60S subunit assembly^5,40,41^. For example, translating ribosomes that are stalled in response to small molecules, peptides, or antibiotics that bind to the NPET display a distorted PTC incapable of catalyzing peptide bond formation^4^. Furthermore, defects in bacterial ribosomes lacking a 5S RNP can be partially rescued by the binding of a macrolide antibiotic, which binds to the NPET^42^. Our data and proposed model suggest that this communication between the NPET and other ribosomal domains can indeed occur during ribosome assembly, and that failure to do so can halt at least three stages of maturation.

In this study, we set out to gain a deeper understanding of how construction of a functional center such as the NPET could fit into the known hierarchy of 60S subunit assembly. Since crucial features of the NPET lie deep within the core of the pre-60S subunit, cells need to be able to detect such subtle but impactful defects in NPET assembly. By coupling NPET construction with the stability of critical rRNA helices, cells might easily identify and terminate assembly of particles harboring a defective NPET. This study opens up the possibility that construction of other functional centers in the eukaryotic ribosome may be regulated in a similar fashion and lays the foundation for future study of mutant 60S subunits using cryo-EM.

## Methods

### Construction of mutant plasmids

Plasmids containing each AF gene were obtained from the Yeast Genomic Tiling Collection (Open Biosystems), and used as templates in a multi-step PCR protocol for inserting unique restriction sites upstream and downstream of the gene^43^. PCR products were digested using restriction enzymes and ligated into pRS315 containing the *LEU2* gene. Mutagenesis of ORFs was performed using the Quickchange Site-directed Mutagenesis Kit (Agilent Technologies). Correct mutations were confirmed by sequencing (Genewiz).

### Generation of yeast strains

All genomic epitope tags, *GAL*-promoter fusions, knockouts, or C-terminal truncations were constructed according to standard methods^44^. The *nog1*∆*C rei1*∆*C reh1*∆*C* triple truncation strain was made by truncating one assembly factor gene at a time. A strain conditional for expression of only *rpl4*∆*63–87* was constructed as follows. First the endogenous promoter of *RPL4A* was replaced with the conditional *GAL* promoter, plus the HA-epitope in-frame with the *RPL4A* ORF. Then, *RPL4B* was knocked out by replacement of the ORF with KanMX. This *GAL-HA-RPL4A rpl4b*Δ strain was transformed with a *LEU2* plasmid pRS315 containing *rpl4a*Δ*63–87*. Arx1-GFP strains were a generous gift from Arlen Johnson (University of Texas at Austin). All strains and plasmids are available upon request. (Supplementary Tables 2 and 3).

### Growth assays

Yeast strains were grown to an OD_600_ of ~0.5 in permissive conditions and then serially diluted out to 10^−5^ -fold before pipetting 10–15 μL of each dilution onto appropriate galactose- or glucose-containing solid media. Plates were checked daily after being incubated at 30 or 16 °C.

### Sucrose density gradient analysis

Preribosomes, ribosomes, and polyribosomes were fractionated from 40 OD_254_ units of yeast whole-cell extracts on 7–47% (w/v) sucrose gradients^30,45^. Five milligrams of cycloheximide (Sigma) was added to 150 ml of culture 20 min before harvesting the cells. Extracts were made using lysis buffer (10 mM Tris-HCl pH 7.5, 0.1 M NaCl, 30 mM MgCl_2_, 50 μg/ml cycloheximide, 200 μg/ml heparin, and 0.2% diethyl pyrocarbonate). Cells were vortexed eight times for 30 s with glass beads (0.5 mm diameter, Biospec Products), kept on ice in between vortexing, and clarified by two consecutive centrifugations. Forty OD_254_ units of whole-cell extracts were layered on 7–47% (w/v) sucrose gradients, and preribosomes, ribosomes, and polyribosomes were fractionated according to the manufacturer’s protocol (Teledyne ISCO). The sucrose gradients were spun in an ultracentrifuge for 4 h. A Foxy R1 density gradient fractionator was used to fractionate and analyze gradients with continuous monitoring at OD_254_.

### Fluorescence microscopy

The intracellular location of preribosomes or AFs was determined as follows: 35 mm glass bottom microwell dishes (MaTek) were coated with concanavalin A (Sigma) and allowed to dry for 40 min. Cells expressing GFP-tagged uL23 or Arx1-GFP were grown to an OD_600_ of 0.3–0.5 before plating 10 μL onto the glass bottom of the MaTek dish and incubating at room temperature for 20 min. Plates were then washed once with 1 mL of appropriate media before being overlaid with 2 mL of media and then imaged using a Ziess LSM 880 confocal microscope. Images were processed using Fiji^46^.

### Analysis of pre-rRNA processing

Steady-state levels of pre-rRNAs were assayed using northern blot and primer extension anaysis^47^. Ten milliliter of cells were harvested, frozen, and RNA was extracted using phenol. Five microgram of RNA was used for primer extension reactions or loaded onto a formaldehyde/MOPS agarose gel for northern blotting. 32Pγ -ATP radiolabeled oligonucleotide probes for specific pre-rRNAs were used in primer extension reactions and for hybridization in northern blots. For northern hybridization of small molecular weight RNAs (7, 6, 5.8, and 5S), RNA samples were mixed with an equal volume of sample buffer (0.1× TBE buffer, 10 M urea, 0.1% xylene cyanol, 0.1% bromophenol blue) and subjected to electrophoresis on a 5% acrylamide/7 M urea gel for 4 h at 120 mA. Following electrophoresis, gels were electroblotted to a Nytran N membrane (GE Healthcare Life Sciences) using a Trans-Blot Plus Cell (Biorad), hybridized with an end-labeled oligonucleotide, washed, and exposed to X-ray film.

### Affinity purification and analysis of preribosomes by silver staining, western blotting, and iTRAQ mass spectrometry

The protein composition profiles of affinity-purified preribosomes was analyzed by SDS-PAGE (4–20% Tris-glycine and 4–12% Bis-Tris, Thermo Fisher Scientific) followed by silver staining^33^. Western blotting tested incorporation of specific proteins into preribosomes^30^. Preribosomes were purified by lysing frozen cells using acid-washed glass beads and TNM150 buffer consisting of 50 mM Tris HCl pH 7.5, 150 mM NaCl, 1.5 mM MgCl_2_, 0.1% NP-40 (Sigma–Aldrich), and 5 mM βME. Following lysis, lysates were incubated with IgG-conjugated Dynabeads while rocking for 1 h at 4 °C. Dynabeads were washed with TNM150 buffer (for iTRAQ, this step excluded NP-40). Samples were eluted using TEV protease while rocking for 1 h at room temperature, then precipitated with 10% TCA and washed with 100% cold acetone. Purified samples were sent to the Penn State Hershey Core Research Facilities for trypsin digestion and 4-plex labeling with iTRAQ reagents 114, 115, 116, 117 (Applied Biosystems).

### Purification of preribosomes for cryo-EM

Preparation of Nog2-associated particles described here required 20 L of cells grown to an OD_600_ of 0.8–1.0 in appropriate media. Frozen cell pellets were lysed using TAP lysis buffer consisting of 50 mM Tris-HCl pH 7.5, 100 mM NaCl, 10 mM MgCl_2_, and 0.075% NP-40 (Sigma–Aldrich). Lysates were incubated with IgG-conjugated Dynabeads while rocking for 1 h at 4 °C. Dynadeads were washed using TAP lysis buffer three times and then washed twice using TEV cleavage/resuspension buffer (TAP lysis buffer with NP-40 excluded). Samples were eluted using TEV protease while rocking for 1 h at room temperature. The eluate was then added to a 100 K centrifugal filter (Millipore), prewashed with TEV cleavage-resuspension buffer, and spun at 14,000 × *g* for 5 min at 4 °C. The flow through was discarded and the sample was recovered by inverting the filters and spinning again at 1000 × *g* for 2 min before being stored at −80 °C until preparation for cryo-EM.

### Cryo-EM data acquisition

Vitrified specimens were prepared by adding 4 μl samples of *nog1*Δ*C rei1*Δ*C reh1*Δ*C* or *rpl4*∆*63–87* particles at a concentration of ~150 nM to a glow-discharged holey carbon grid (Quantifoil R1.2/1.3) covered with a freshly made thin carbon film. Grids were blotted for 0.5 s and plunge-frozen into liquid ethane using an FEI Vitrobot Mark IV (4 °C and 100% humidity). The cryo-grids were initially screened at a nominal magnification of 92,000× in an FEI Talos Arctica microscope, operated with an acceleration voltage of 200 kV. Good-quality grids were transferred to an FEI Titan Krios electron microscope that was operating at 300 kV. Images were recorded using a K2 Summit direct electron detector (Gatan) in counting mode at a nominal magnification of ×105,000 (for the *rpl4*∆*63–87* sample) and ×130,000 (for the *nog1*Δ*C rei1*Δ*C reh1*Δ*C* sample), respectively (pixel sizes on the object scale are 1.373 and 1.052 Å, respectively), with the defocus ranging from −1.0 to −2.0 μm. Coma-free alignment was manually optimized and parallel illumination was verified before data collection. All micrographs with the K2 camera were collected semi-automatically by SerialEM^48^ under low-dose conditions. Each micrograph was dose-fractionated to 32 frames with a dose rate of ~10.0 counts per physical pixel per second for a total exposure time of 6.4 s.

### Cryo-EM data processing

Original image stacks were summed and corrected for drift and beam-induced motion at the micrograph level using the MotionCor2 program^49^. The SPIDER^50^ software was used for micrograph screening. The contrast transfer function parameters of each micrograph were estimated by Gctf (Zhang, K. 2016)^51^. All 2D and 3D classification and refinement were performed with RELION3.0 (Zivanov, J. et al. 2018)^52^. The local resolution map was estimated using ResMap^53^.

For the *rpl4*∆*63–87* mutant sample, a total of 5003 micrographs were collected and 883,649 particles were picked for a cascade 2D and 3D classification with a binning factor of two. About 57% of particles were removed during several rounds of 2D and 3D classification, and 382,748 particles were split into ten classes during the final round of 3D classification (Supplementary Fig. 2). After the final round of 3D classification, a total of 103,319 particles were applied for high-resolution refinement (without binning), resulting in a 3.12 Å map (gold-standard FSC 0.143 criteria) (Supplementary Fig. 2c). A total of 47,025 particles were applied for high-resolution refinement (without binning), resulting in a 3.22 Å map (Supplementary Fig. 2d). In addition to these two states, class R3 is similar to the state B of Nsa1-particles^16^ and class R4 is similar to the state E of Nsa1-particles but with already assembled 5 S RNP (in the premature rotated state). Classes R5-R7 also have well resolved structural features; but they lack densities for major domains of rRNA, suggesting that they are likely turnover products (Supplementary Fig. 3a–e).

For the *nog1*Δ*C rei1*Δ*C reh1*Δ*C* mutant sample, a total of 9115 micrographs were collected and 1,009,266 particles were picked for cascade 2D and 3D classification with a binning factor of four. About 67% of particles were removed during three rounds of 2D classification, and 332,827 particles were split into ten classes during the first round of 3D classification, with a map of the premature 60 S ribosomal subunit (EMD-6615) (low-pass filtered to 60 Å) as the initial model. Based on the map features (the presence of ITS2 and rotation of the 5S RNP), classes were combined into two groups and were subjected to a second round of 3D classification. The first group, with the 5 S RNP in premature state, was classified into ten classes. A majority of them (62.8% particles, class N1) represent a state very similar to the state 1 of wild-type Nog2-particles^17^. A total of 89,239 particles were applied for high-resolution refinement (without binning), resulting in a 3.00 Å map (Supplementary Fig. 7c). The second group, with the 5S RNP in mature-like position, containing 169,432 particles, was also subjected to a second round of 3D classification into ten classes. Around 62.6% of these particles are mature or mature-like 60S subunits.

### Model building and refinement

An atomic model of wild-type Nog2-TAP state 1 (PDB: 3jct)^17^ was used as the initial template for modelling. The models of the rRNAs (25S, 5.8S, 5S, ITS2 RNA) were docked into the density map manually using UCSF Chimera^54^. For RP and AF modelling, structures of individual proteins were separately fitted into their density by rigid-body docking. After the initial fitting, the entire chains of rRNAs and proteins were manually checked and adjusted with COOT^55^. The atomic model of classes R1 and R2 of the *rpl4*∆*63–87* mutant particles were further refined against the density map first by real-space refinement (phenix.real_space_refine)^56^ in PHENIX^57^, with secondary structure restraints, geometry restraints and RNA-specific restraints applied. After refinement, alternating rounds of manual model adjustment using COOT and model refinement using PHENIX were applied. The atomic models were cross-validated according to standard procedures^58,59^. Specifically, the coordinates of the final model were randomly displaced by 0.2 Å using the PDB tools of Phenix. The displaced model was refined against the Half1 map (produced from a half set of all particles during refinement by RELION). The refined model from Half1 map was compared with the maps of Half1, Half2 in Fourier space to produce two FSC curves, FSC_work_ (model versus Half1 map) and FSC_free_ (model versus Half2 map), respectively (Supplementary Fig. 2c, d). Another FSC curve between the refined model from Half1 and the final density map (model versus full) from all particles was also produced. As indicated by these curves, the agreement between FSC_work_ and FSC_free_ (no large separation) indicated that the model was not overfitted. MolProbity^60^ (http://molprobity.biochem.duke.edu/) was used to evaluate the final model, and final statistics of the model are provided in Supplementary Table 1.

### Statistics and reproducibility

The experiment shown in Fig. 2a was performed three times. The experiment shown in Fig. 4a was performed three times. The experiment shown in Fig. 4b was performed twice. The experiment shown in Fig. 5a was performed three times. Experiments shown in Fig. 7a, b were performed three times. The experiment shown in Supplementary Fig. 1a was performed three times. The experiment shown in Supplementary Fig. 1b was performed twice. The experiments shown in Supplementary Fig. 6b, c were performed three times. The experiment shown in Supplementary Fig. 10a was performed twice.

### Reporting summary

Further information on research design is available in the Nature Research Reporting Summary linked to this article.

## Supplementary information

Supplementary Information

Peer Review

Reporting Summary

## Data Availability

The data that support this study are available from the corresponding authors upon reasonable request. The cryo-EM density maps of the R1, R2 classes of the *rpl4∆63–87* mutant particles and the N1, N2, N3, N4 classes of the *nog1*Δ*C rei1*Δ*C reh1*Δ*C* mutant particles have been deposited in the Electron Microscopy Data Bank (EMDB) under accession numbers EMD-30170, EMD-30174, EMD-30172, EMD-30173, EMD-30175, and EMD-30176, respectively; and the atomic models of the R1 and R2 classes of the *rpl4*∆*63–87* mutant particles have been deposited in the Protein Data Bank (PDB) under accession numbers 7BT6 and 7BTB, respectively. Source data are provided with this paper.

## Source data

Source Data
