# Supplementary material for: Structural insights into assembly of the ribosomal nascent polypeptide exit tunnel
Source: Nat Commun. 2020 Oct 9;11:5111. doi: 10.1038/s41467-020-18878-8 (PMC7547690; doi:10.1038/s41467-020-18878-8)
Supplement: Supplementary file 1 — Supplementary Information [file 41467_2020_18878_MOESM1_ESM.pdf]

SUPPLEMENTARY INFORMATION FOR

Structural insights into assembly of the ribosomal nascent polypeptide exit tunnel

Wilson et al.

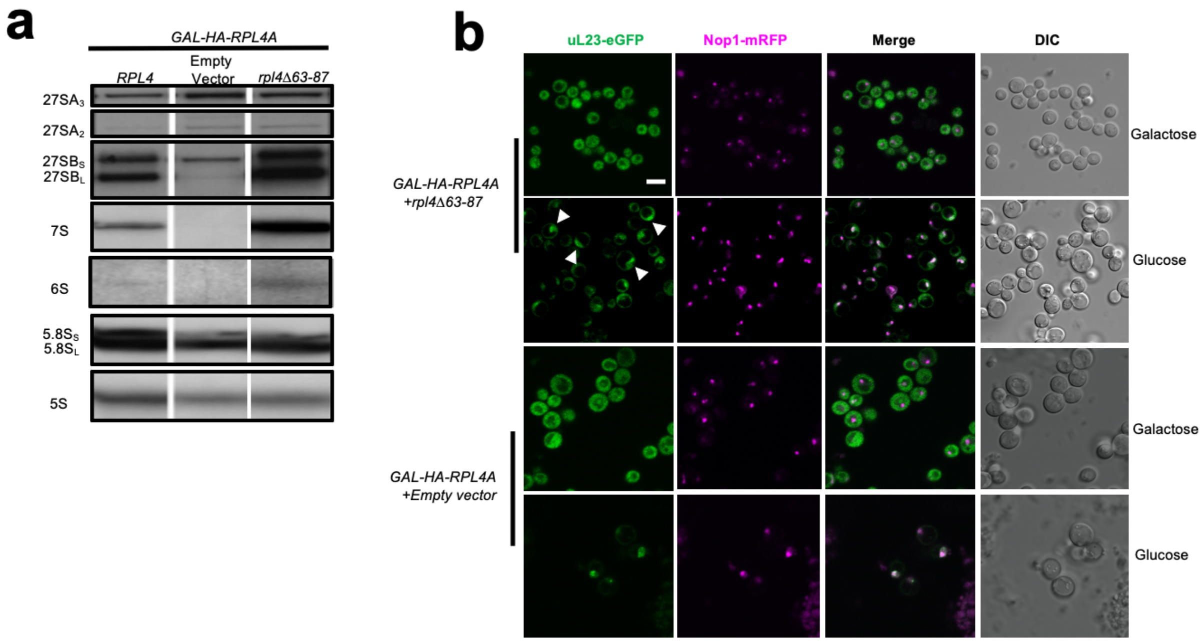

Supplementary Figure 1. Mutant *rpl4Δ63-87* pre-60S subunits fail nuclear export. (a) 27S and 7S pre-rRNAs accumulate in the *rpl4Δ63-87* mutant. Cells were shifted from galactose to glucose media for 17 h. before extracting whole cell RNA. Steady-state levels of pre-rRNAs from each strain were then assayed by primer extension and gel electrophoresis. (b) Pre-60S subunits accumulate in the nucleoplasm of the *rpl4Δ63-87* mutant, after being shifted from galactose to glucose media for 17 h. to deplete wild-type uL4. In contrast, in the absence of the entire uL4 protein (empty vector), pre-ribosomes accumulate in the nucleolus. uL23-eGFP (green) tracks pre-60S particles and Nop1-mRFP (magenta) marks the nucleolus. Scale bar: 5μm.

Supplementary Figure 2

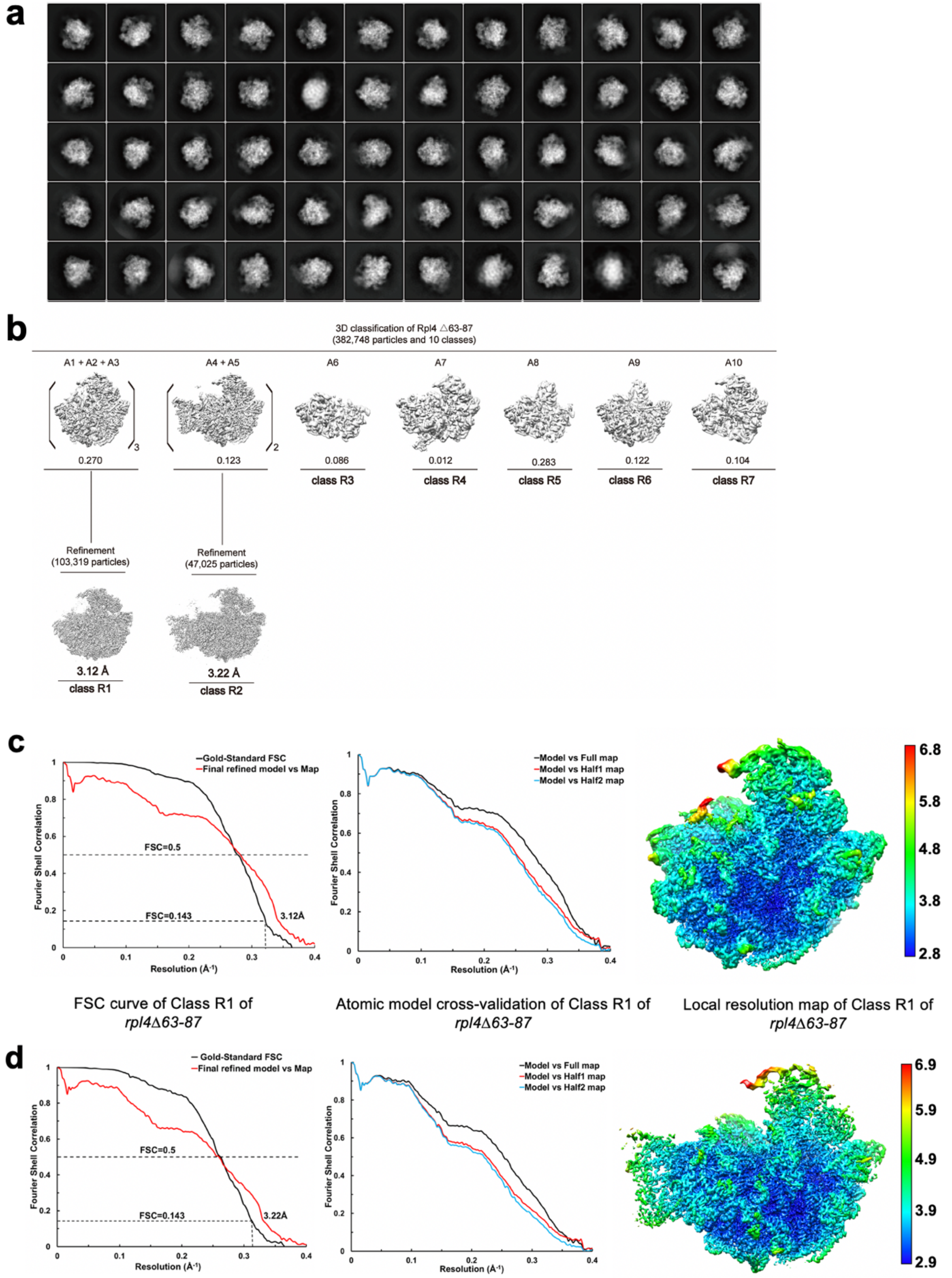

FSC curve of Class R1 of *rpl4Δ63-87*

Atomic model cross-validation of Class R1 of *rpl4Δ63-87*

Local resolution map of Class R1 of *rpl4Δ63-87*

FSC curve of Class R2 of *rpl4Δ63-87*

Atomic model cross-validation of Class R2 of *rpl4Δ63-87*

Local resolution map of Class R2 of *rpl4Δ63-87*

Supplementary Figure 2. Cryo-EM data processing of *rpl4∆63-87* Nog2 particles.

(a) Representative 2D class averages of *rpl4∆63-87* mutant Nog2 particles. (b) Flow chart for 3D classification. (c) Fourier shell correlation (FSC) curves for the final 3D density map of class R1 after RELION-based post-processing (dark, gold-standard FSC), and for the cross-examination between the final atomic model and the 3D density map (red, final refined model versus map) (left panel). At an FSC 0.143 cut-off, the overall resolution for the map is 3.12 Å. The FSC 0.5 cut-off is also shown. FSC curves for the atomic model cross-validation (middle panel). See Methods for details. Local resolution map for class R1 (right). (d) Fourier shell correlation (FSC) curves for the final 3D density map of class R2 after RELION-based post-processing (dark, gold-standard FSC), and for the cross-examination between final atomic model and the 3D density map (red, final refined model versus map) (left panel). At an FSC 0.143 cut-off, the overall resolution for the map is 3.22 Å. The FSC 0.5 cut-off is also shown. FSC curves for the atomic model cross-validation (middle panel). See Methods for details. Local resolution map for class R2 (right).

Supplementary Figure 3

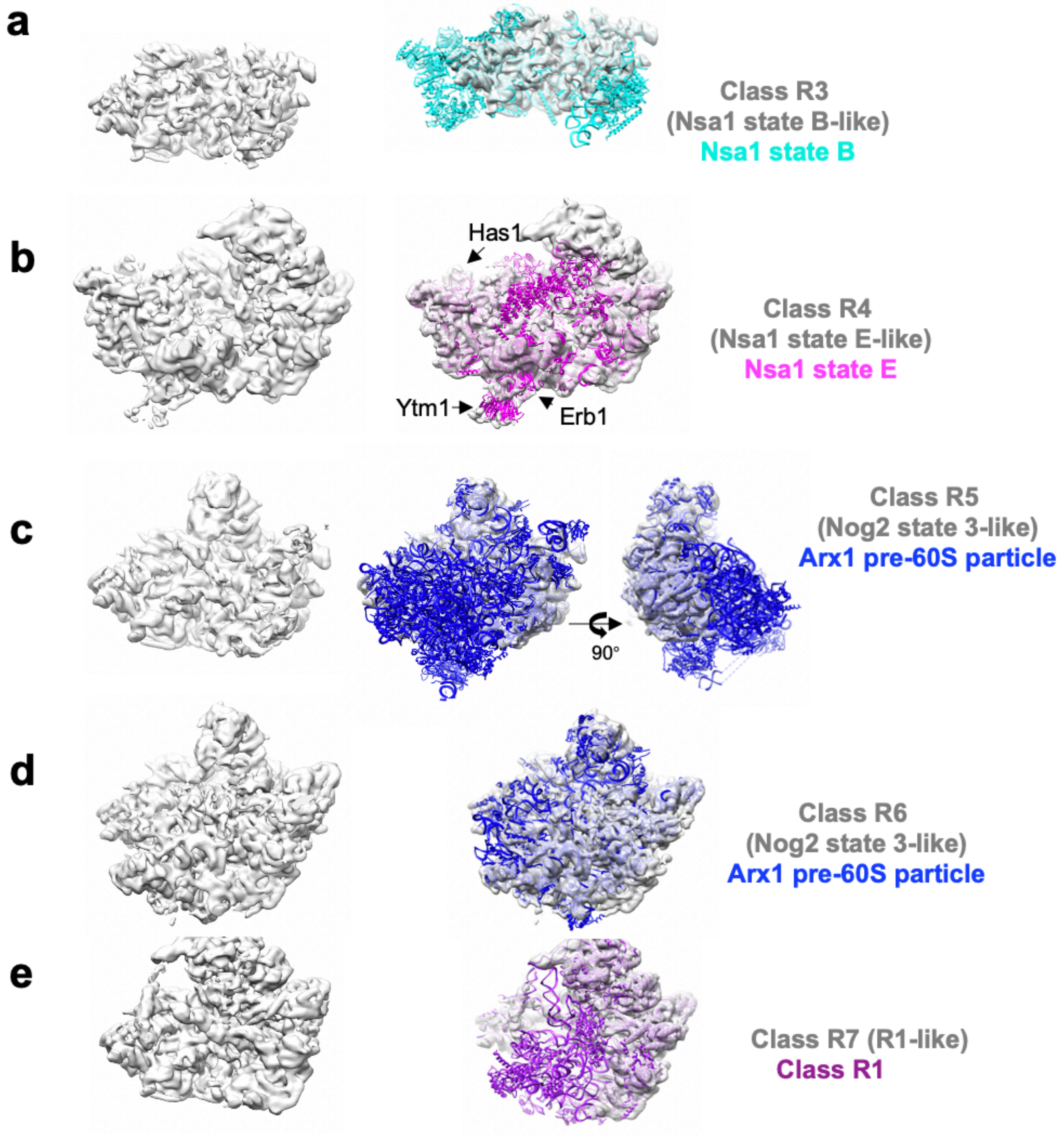

Supplementary Figure 3. Nog2 particles purified from the *rpl4Δ63-87* mutant. (a) Densities of class R3 (gray) fitted with the atomic model of the Nsa1 state B particle (cyan) (PDB: 6EM4). (b) Densities of class R4 (gray) fitted with the atomic model of

Nsa1 state E (purple) (PDB: 6ELZ). (c and d) Densities of classes R5 and R6 (gray) fitted with the atomic model of the Arx1 particle (blue) (PDB: 4V7F). (e) Densities of class R7 (gray) fitted with the atomic model of class R1 state (purple).

Supplementary Figure 4

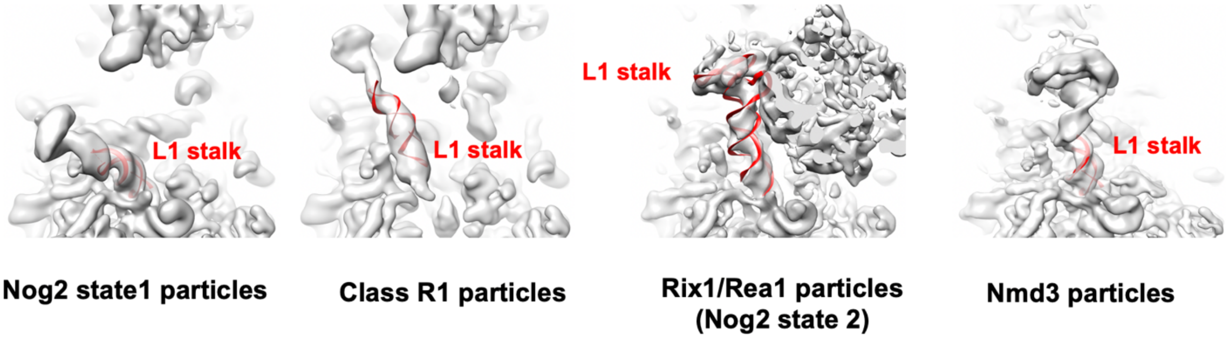

Supplementary Figure 4. Class R1 exhibits a deflected L1 stalk. Cryo-EM density maps of four assembly intermediates, wild-type Nog2 state1, mutant class R1, wild-type Rix1/Rea1 state (Nog2 state 2), and the wild-type Nmd3 state are displayed in transparent surface representation, superimposed with their own atomic model. rRNA for the L1 stalk is shown in red.

Supplementary Figure 5

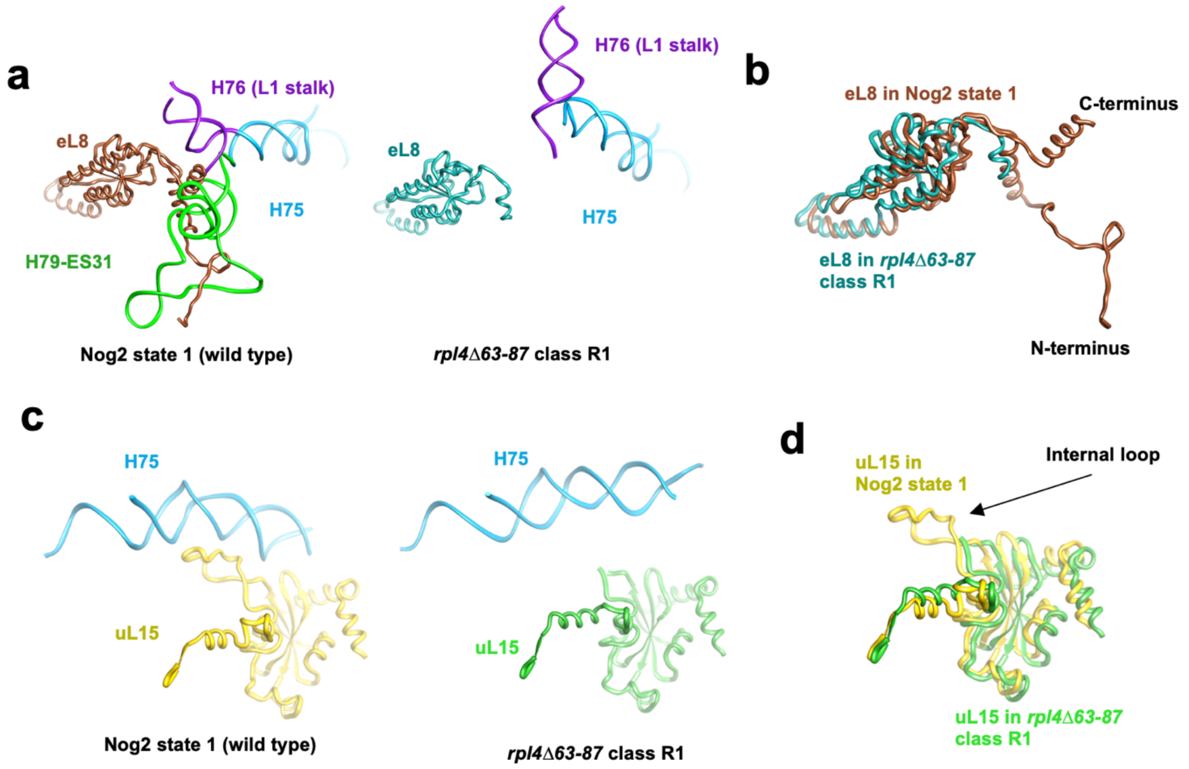

Supplementary Figure 5. Defects in H75 are communicated to eL8 and uL15. (a) RP eL8 in wild-type Nog2 state 1 particles (left) shown in relation to the 3-way junction of H75 (blue), H76 (light purple), and H79 (green). On the right, the same components are shown in class R1 of the *rpl4Δ63-87* mutant particles. (b) eL8 from wild-type Nog2 state 1 particles (brown) overlaid with eL8 from class R1 (dark teal). In class R1, both the N- and C- termini are flexible. (c) RP uL15 (yellow) in wild-type Nog2 state 1 particles (left) shown in relation to H75 (blue). On the right, the same components are shown in class R1 of the *rpl4Δ63-87* mutant particles. (d) uL15 from wild-type state 1 particles (yellow) overlaid with uL15 from *rpl4Δ63-87* class R1 particles (light green). In class R1, the internal loop of uL15 is flexible.

Supplementary Figure 6

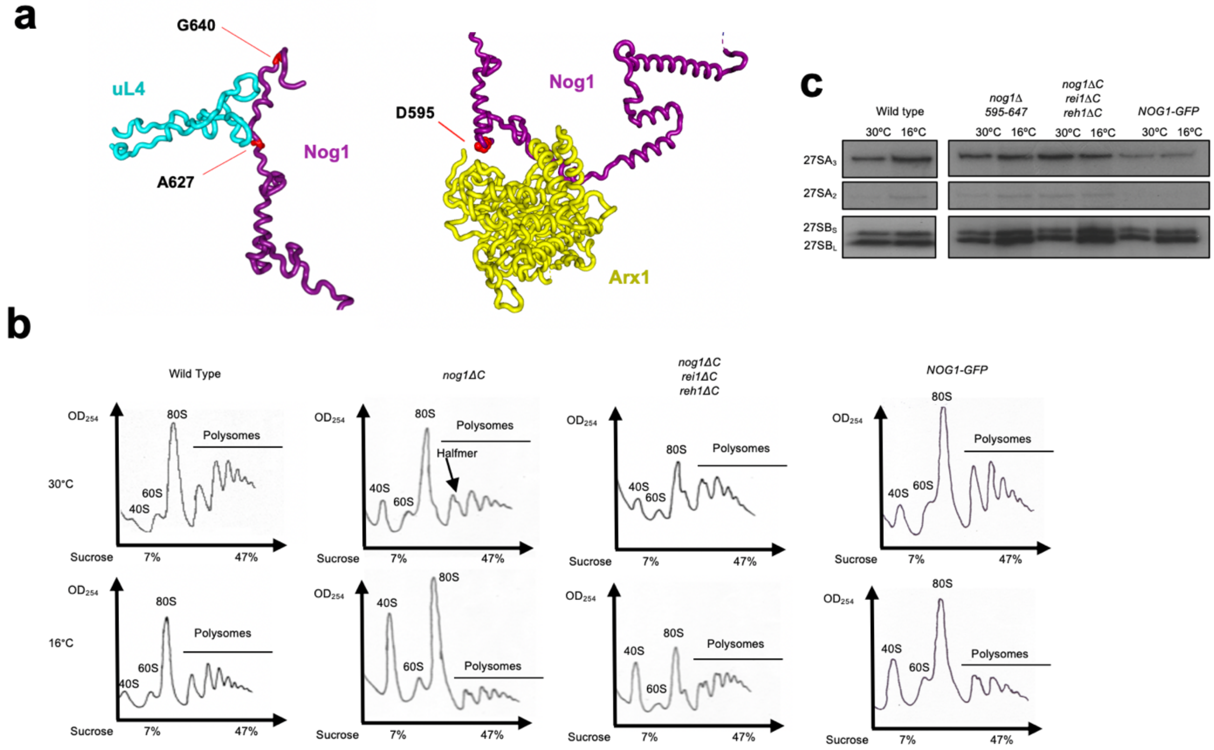

Supplementary Figure 6. Truncation of the Nog1 CTD causes cold-sensitive defects. (a) PyMol model of the Nog1 CTD (purple) in the context of the uL4 internal loop (cyan) (left) and Arx1 (yellow) (right). Red residues mark endpoints of respective truncations. (b) Sucrose gradient fractionation of whole-cell lysate collected from *nog1* mutants grown at 30ºC or shifted to 16ºC for five hours. (c) Primer extension of RNA extracted from *nog1* mutants grown at 30ºC or shifted to 16ºC for five hours.

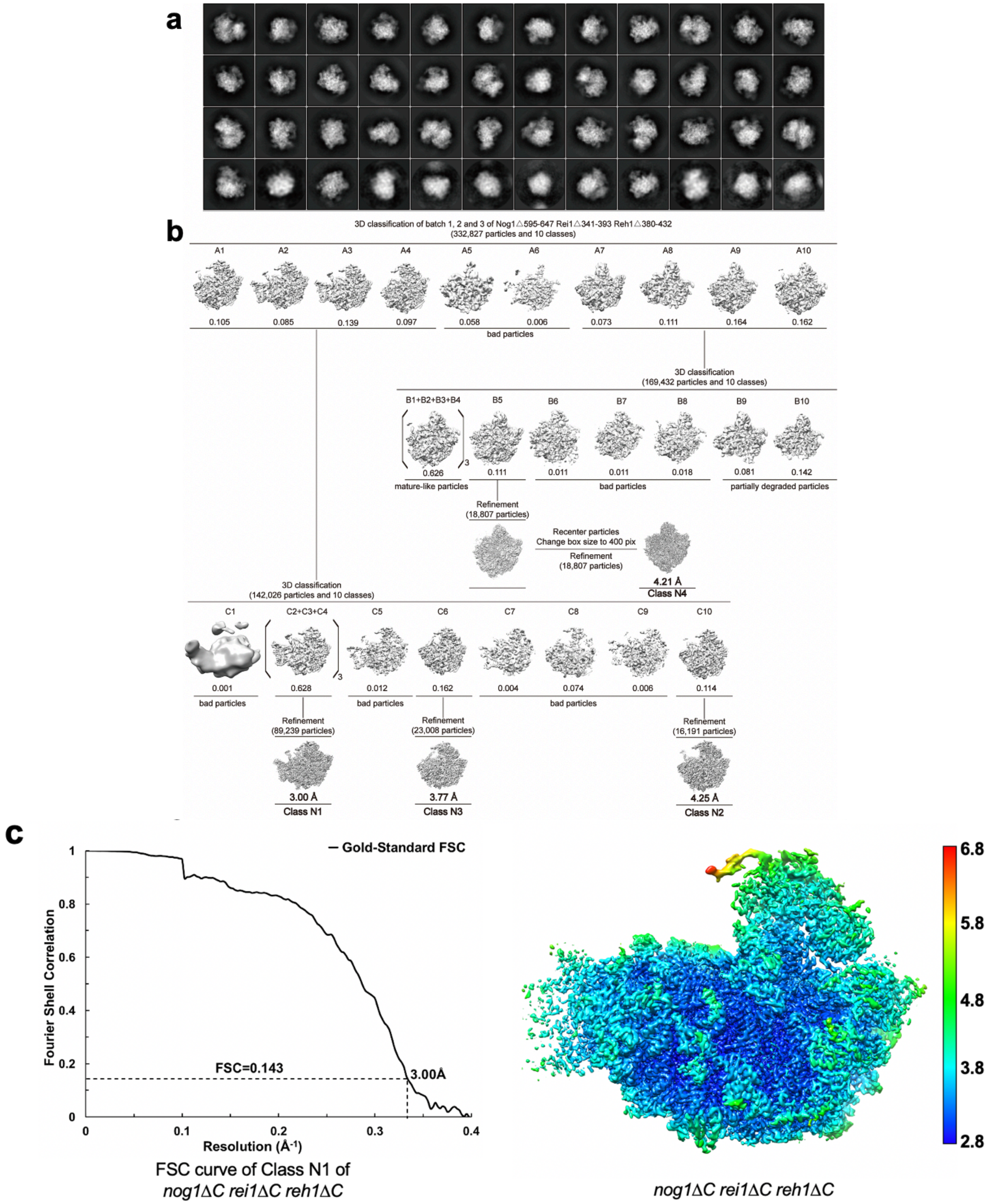

Supplementary Figure 7. Cryo-EM data processing of *nog1ΔC rei1ΔC reh1ΔC* particles. (a) Representative 2D class averages of *nog1ΔC rei1ΔC reh1ΔC* mutant

Nog2 particles. (b) Flow chart for 3D classification. (c) FSC curve and local resolution map for class N1.

a

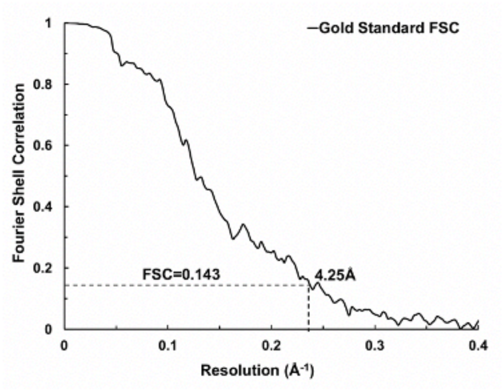

FSC curve of Class N2 of
nog1ΔC rei1ΔC reh1ΔC

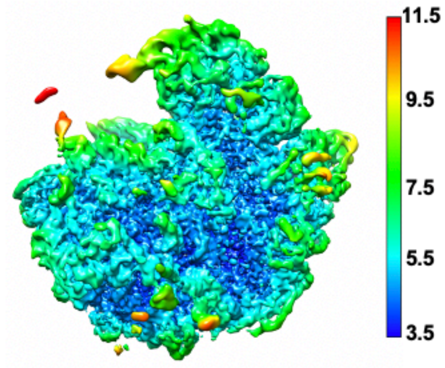

Local resolution map of Class N2 of
nog1ΔC rei1ΔC reh1ΔC

b

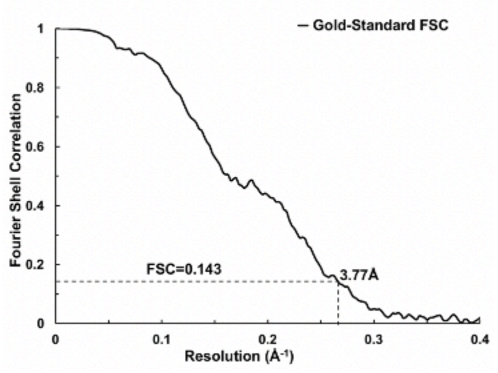

FSC curve of Class N3 of
nog1ΔC rei1ΔC reh1ΔC

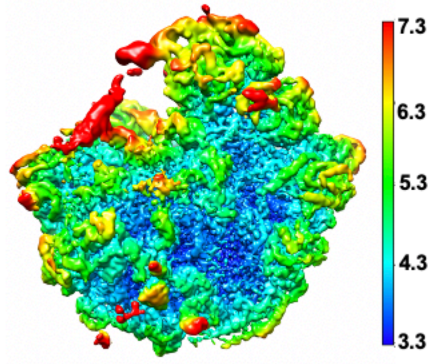

Local resolution map of Class N3 of
nog1ΔC rei1ΔC reh1ΔC

c

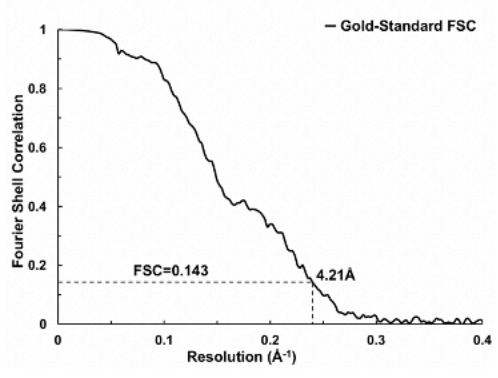

FSC curve of Class N4 of
nog1ΔC rei1ΔC reh1ΔC

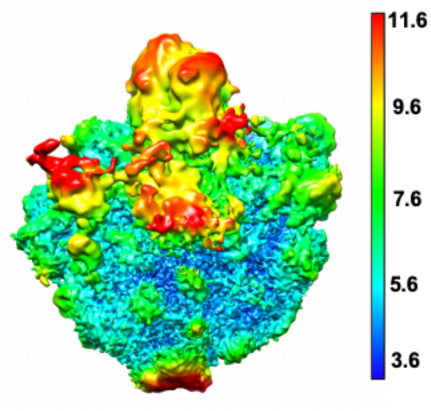

Local resolution map of Class N4 of
nog1ΔC rei1ΔC reh1ΔC

Supplementary Figure 8. FSC curves and local resolution maps of classes N2-N4.

(a) FSC curve (left) and local resolution map (right) of class N2. (b) FSC curve (left) and

local resolution map (right) of class N3. (c) FSC curve (left) and local resolution map (right) of classN3. (C) FSC curve (left) and local resolution map (right) of class N4.

a

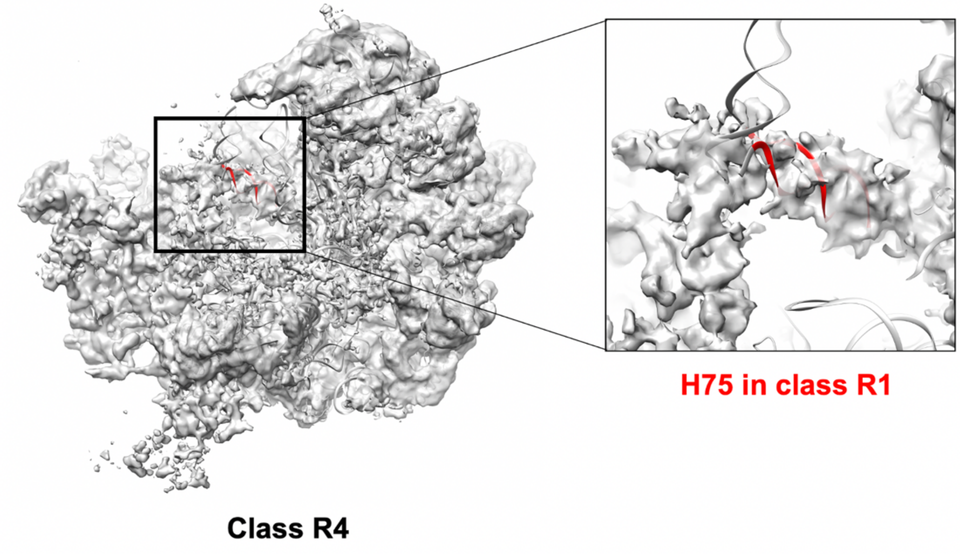

b

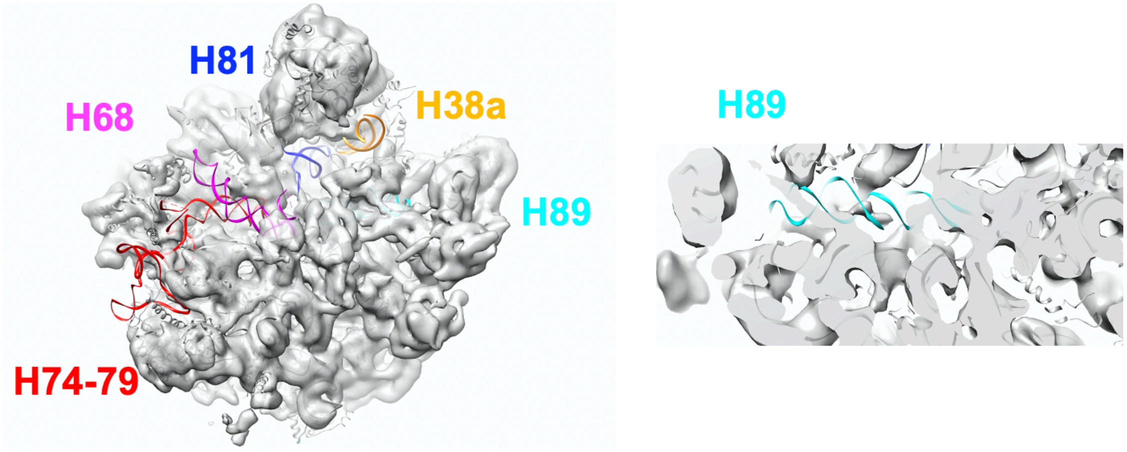

Class R6 densities aligned with the Arx1 particle atomic model (Nog2 state 3)

Supplementary Figure 9. Classes R4 and R6 display aberrant rRNA conformations. (a) Class R4 of the *rpl4Δ63-87* mutant is aligned with the atomic model of class R1. H75 exhibits an aberrant conformation similar to the H75 observed in class R1 (red). (b) Densities for class R6 from the *rpl4Δ63-87* mutant are aligned with the atomic model of

the Arx1 particle (PDB: 5APO), which resembles Nog2 state 3 particles. Exposed atomic models for rRNA helices 38a (orange), 68 (magenta), 74-79 (red), 81 (blue), and 89 (cyan) represent flexible helices.

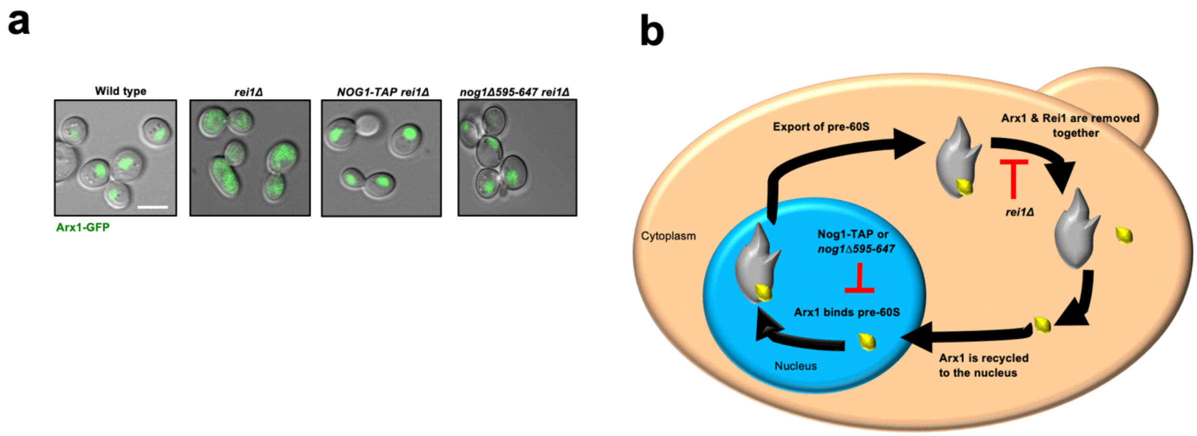

Supplementary Figure 10. The Nog1 CTD is required to recruit Arx1. (a) Confocal microscopy of Arx1-GFP cells in different genetic backgrounds. Scale bar: 5μm. (b) Model representing the effect that different AF mutations have on the cycle of Arx1 (yellow) binding and release from pre-60S subunits.

Supplementary Table 1 | Cryo-EM data collection, refinement and validation statistics

	**Class R1**	**Class R2**
EM equipment	FEI Titan krios	FEI Titan krios
Voltage(kV)	300	300
Detector	Gatan K2	Gatan K2
Particles	103,319	47,025
Pixel size (Å)	1.373	1.373
Defocus range (µm)	1.0-2.0	1.0-2.0
Electron dose (e^-/Å^2)	64 (32 frames)	64 (32 frames)
**Model composition**		
Peptide chains	52	55
Protein residues	8131	9532
RNA chains	3	4
RNA bases	3241	3402
**Refinement**		
Resolution (Å))	3.12	3.22
Map sharpening B-factor (Å^2)	-59	-61
Refinement package	Phenix.real_space_refine	Phenix.real_space_refine
Model versus map resolution(Å)(FSC=0.5)	3.55	3.84
**Model-map CC**		
CC (mask)	0.85	0.84
CC (box)	0.79	0.73
CC (peaks)	0.77	0.71
CC (volume)	0.84	0.82
**R.m.s. deviations**		
Bond lengths (Å)	0.008	0.006
Bond angles (°)	1.046	1.010
C-beta deviation	0.00	0.00
CaBLAM outliers	5.29	5.83
**Validation**		
Molprobity score	1.71	1.82
Clash score	3.71	4.99
Rotamer outliers(%)	0.89	0.40
**Ramachandan plot**		
Favored (%)	90.00	89.86

Strain	Genotype	Source
JWY11849	MATa his3Δ1 leu2Δ0 met15Δ0 ura3Δ0 nog1::Nog1-GFP HIS3 nog2::Nog2-TAP URA3	This study
JWY11851	MATa his3Δ1 leu2Δ0 met15Δ0 ura3Δ0 nog1::Nog1-GFP HIS3 nop7::Nop7-TAP URA3	This study
JWY11848	MATa his3Δ1 leu2Δ0 met15Δ0 ura3Δ0 rei1::KANMX6 arx1::Arx1-GFP HIS3	Hung and Johnson, 2006
JWY11858	MATa his3Δ1 leu2Δ0 met15Δ0 ura3Δ0 rei1::KANMX6 arx1::Arx1-GFP HIS3 nog1::Nog1-TAP URA3	This study
JWY11863	MATa ura3-52 trp1-1 lys2-801 his3-Δ200 leu2-Δ1 l4b::KANMX6 l4a::GAL-HA3-RPL4A TRP1 nog2::Nog2-TAP URA3	This study
JWY11865	MATa ura3-52 trp1-1 lys2-801 his3-Δ200 leu2-Δ1 l4b::KANMX6 l4a::GAL-HA3-RPL4A TRP1 nog2::Nog2-TAP URA3 pRS315_L4ΔA63-87 LEU2	This study
JWY11869	MATa ura3-52 trp1-1 lys2-801 his3-Δ200 leu2-Δ1 l4b::KANMX6 l4a::GAL-HA3-RPL4A TRP1 pRS315_L4Δ63-75 LEU2	This study
JWY 11881	MATa ura3-52 trp1-1 lys2-801 his3-Δ200 leu2-Δ1 l4b::KANMX6 l4a::GAL-HA3-RPL4A TRP1 pRS315_L4A LEU2 pRS316_Nop1-mRFP L25-eGFP URA3	This study
JWY11883	MATa ura3-52 trp1-1 lys2-801 his3-Δ200 leu2-Δ1 l4b::KANMX6 l4a::GAL-HA3-RPL4A TRP1 pRS315 LEU2 pRS316_Nop1-mRFP L25-eGFP URA3	This study
JWY11885	MATa ura3-52 trp1-1 lys2-801 his3-Δ200 leu2-Δ1 l4b::KANMX6 l4a::GAL-HA3-RPL4A TRP1 pRS315_L4AΔ63-75 LEU2 pRS316_Nop1-mRFP L25-eGFP URA3	This study
JWY11887	MATa ura3-52 trp1-1 lys2-801 his3-Δ200 leu2-Δ1 l4b::KANMX6 l4a::GAL-HA3-RPL4A TRP1 pRS315_L4A63-87 LEU2 pRS316_Nop1-mRFP L25-eGFP URA3	This study
JWY11899	MATa ura3-52 trp1-1 lys2-801 his3-Δ200 leu2-Δ1 arx1::Arx1-GFP HIS3 rei1::KANMX6	This study
JWY11937	MATa ura3-52 trp1-1 lys2-801 his3-Δ200 leu2-Δ1 nog1::Nog1Δ595-647 KANMX nog2::Nog2-TAP URA3	This study
JWY11939	MATa ura3-52 trp1-1 lys2-801 his3-Δ200 leu2-Δ1 nog1::Nog1Δ595-647 KANMX nmd3::Nmd3-TAP URA3	This study
JWY11941	MATa ura3-52 trp1-1 lys2-801 his3-Δ200 leu2-Δ1 nog1::Nog1Δ595-647 KANMX rei1::Rei1Δ341-393 HIS3 reh1::Reh1Δ380-432 TRP1 nop7::Nop7-TAP URA3	This study
JWY11943	MATa ura3-52 trp1-1 lys2-801 his3-Δ200 leu2-Δ1 nog1::Nog1Δ595-647 KANMX rei1::Rei1Δ341-393 HIS3 reh1::Reh1Δ380-432 TRP1 nog2::Nog2-TAP URA3	This study
JWY11945	MATa ura3-52 trp1-1 lys2-801 his3-Δ200 leu2-Δ1 nog1::Nog1Δ595-647 KANMX rei1::Rei1Δ341-393 HIS3 reh1::Reh1Δ380-432 TRP1 nmd3::Nmd3-TAP URA3	This study
JWY11947	MATa ura3-52 trp1-1 lys2-801 his3-Δ200 leu2-Δ1 nog1::Nog1Δ595-647 KANMX nop7::Nop7-TAP URA3	This study
JWY11959	MATa ura3-52 trp1-1 lys2-801 his3-Δ200 leu2-Δ1 nog1::Nog1Δ627-647 KANMX	This study

JWY11961	MATa ura3-52 trp1-1 lys2-801 his3-Δ200 leu2-Δ1 nog1::Nog1Δ640-647 KANMX	This study
JWY11969	MATa ura3-52 trp1-1 lys2-801 his3-Δ200 leu2-Δ1 nog1::Nog1Δ595-647 KANMX rei1::Rei1Δ341-393 HIS3 reh1::Reh1Δ380-432 TRP1 pRS315_Nop1-mRFP L25eGFP LEU2	This study
JWY11971	MATa ura3-52 trp1-1 lys2-801 his3-Δ200 leu2-Δ1 GAL-NOG2::TRP1 NMD3-TAP::URA3	This study
JWY11973	MATa ura3-52 trp1-1 lys2-801 his3-Δ200 leu2-Δ1 l4b::KANMX6 l4a::GAL-HA3-RPL4A TRP1 nmd3::Nmd3-TAP URA3	This study
JWY11975	MATa ura3-52 trp1-1 lys2-801 his3-Δ200 leu2-Δ1 l4b::KANMX6 l4a::GAL-HA3-RPL4A::TRP1 nmd3::Nmd3-TAP URA3 pRS315_L4AΔ63-87 LEU2	This study
JWY11977	MATa ura3-52 trp1-1 lys2-801 his3-Δ200 leu2-Δ1 l4b::KANMX6 l4a::GAL-HA3-RPL4A::TRP1 pRS315_L4A R69E LEU2	This study
JWY111979	MATa ura3-52 trp1-1 lys2-801 his3-Δ200 leu2-Δ1 l4b::KANMX6 l4a::GAL-HA3-RPL4A::TRP1 pRS315_L4A R73E LEU2	This study
JWY11981	MATa ura3-52 trp1-1 lys2-801 his3-Δ200 leu2-Δ1 l4b::KANMX6 l4a::GAL-HA3-RPL4A::TRP1 pRS315_L4A R69E + R73E LEU2	This study
JWY11982	MATa ura3-52 trp1-1 lys2-801 his3-Δ200 leu2-Δ1 l4b::KANMX6 l4a::GAL-HA3-RPL4A::TRP1 pRS315_L4A 63-68 → Ala	This study
JWY11984	MATa ura3-52 trp1-1 lys2-801 his3-Δ200 leu2-Δ1 l4b::KANMX6 l4a::GAL-HA3-RPL4A::TRP1 pRS315_L4A 69-74 → Ala	This study

Supplementary Table 2. Yeast strain list.

Plasmid	Description	Source
pJW10704	*rpl4∆63-87* LEU2 CEN	11
pJW11869	*rpl4∆63-75* LEU2 CEN	This study
pJW11981	*rpl4R69E, R73E* LEU2 CEN	This study
pJW11982	*rpl4-1* LEU2 CEN	This study
pJW11984	*rpl4-2* LEU2 CEN	This study
pJW10672	*rpl4* LEU2 CEN	11
pRS315	LEU2 CEN	11

Supplementary Table 3. Plasmid list.
