# Supplementary material for: Structural insights into assembly of the ribosomal nascent polypeptide exit tunnel
Source: Nat Commun. 2020 Oct 9;11:5111. doi: 10.1038/s41467-020-18878-8 (PMC7547690; doi:10.1038/s41467-020-18878-8)
Supplement: Supplementary file 3 — Reporting Summary [file 41467_2020_18878_MOESM3_ESM.pdf]

nature research

Corresponding author(s): Ning Gao, John L Woolford, Jr.

Last updated by author(s): Aug 20, 2020

Reporting Summary

Nature Research wishes to improve the reproducibility of the work that we publish. This form provides structure for consistency and transparency in reporting. For further information on Nature Research policies, see Authors & Referees and the Editorial Policy Checklist .

Statistics

For all statistical analyses, confirm that the following items are present in the figure legend, table legend, main text, or Methods section.

n/a	Confirmed	
☐	☒	The exact sample size (n) for each experimental group/condition, given as a discrete number and unit of measurement
☐	☒	A statement on whether measurements were taken from distinct samples or whether the same sample was measured repeatedly
☒	☐	The statistical test(s) used AND whether they are one- or two-sided<br>*Only common tests should be described solely by name; describe more complex techniques in the Methods section.*
☒	☐	A description of all covariates tested
☒	☐	A description of any assumptions or corrections, such as tests of normality and adjustment for multiple comparisons
☐	☒	A full description of the statistical parameters including central tendency (e.g. means) or other basic estimates (e.g. regression coefficient) AND variation (e.g. standard deviation) or associated estimates of uncertainty (e.g. confidence intervals)
☒	☐	For null hypothesis testing, the test statistic (e.g. F, t, r) with confidence intervals, effect sizes, degrees of freedom and P value noted<br>*Give P values as exact values whenever suitable.*
☒	☐	For Bayesian analysis, information on the choice of priors and Markov chain Monte Carlo settings
☒	☐	For hierarchical and complex designs, identification of the appropriate level for tests and full reporting of outcomes
☒	☐	Estimates of effect sizes (e.g. Cohen's d, Pearson's r), indicating how they were calculated

Our web collection on statistics for biologists contains articles on many of the points above.

Software and code

Policy information about availability of computer code

Data collection	Cryo-EM data were recorded using the software Serial EM v3.6, and raw particles were randomly selected using the computer program RELION 3.0; Images for fluorescence microscopy were acquired using ZEN 2 software, blue edition (Zeiss)
Data analysis	Cryo-EM data were processed using MotionCor2 v1.1.0, Gctf v1.06, Chimera v1.11.2, Pymol v2.x, Coot v0.8.7 and Phenix v1.18.2; Protein Pilot 5.0 was used to obtain iTRAQ ratios as an average of all peptides for each protein; Images for fluorescence microscopy were processed using Fiji for Mac OSX (National Institutes of Health).

For manuscripts utilizing custom algorithms or software that are central to the research but not yet described in published literature, software must be made available to editors/reviewers. We strongly encourage code deposition in a community repository (e.g. GitHub). See the Nature Research guidelines for submitting code & software for further information.

Data

Policy information about availability of data

All manuscripts must include a data availability statement. This statement should provide the following information, where applicable:

- Accession codes, unique identifiers, or web links for publicly available datasets
- A list of figures that have associated raw data
- A description of any restrictions on data availability

The cryo-EM density maps of the R1, R2 classes of the rpl4Δ63-87 mutant particles and the N1, N2, N3, N4 classes of the nog1ΔC rei1ΔC reh1ΔC mutant particles have been deposited in the Electron Microscopy Data Bank (EMDB) under accession numbers EMD-30170, EMD-30174, EMD-30172, EMD-30173, EMD-30175 and EMD-30176, respectively; and the atomic models of the R1 and R2 classes of the rpl4Δ63-87 mutant particles have been deposited in the Protein Data Bank (PDB) under accession numbers 7BT6 and 7BTB, respectively. The PDB or EMD files for publicly available cryo-EM structures used in this study are listed as follows: Nsa1 state 2 (6C0F), Nsa1 state C (6EM1), Nsa1 state E (6ELZ), Nog2 state 1 (3JCT), Rix1/Rea1 particle (5FL8), Arx1 particle (5APN), Nmd3 particle (5H4P), and the mature 80S crystal structure of the yeast ribosome (4V88). Yeast strains, plasmids used in this study, and sequences for oligonucleotides are available upon request. Source data for Figs. 2a, 4a, 4b, 5a, 7a, 7b, and Supplementary Figs. 1a, 6c, 6d, and 10a are provided with the paper.

Field-specific reporting

Please select the one below that is the best fit for your research. If you are not sure, read the appropriate sections before making your selection.

- [x] Life sciences
- [ ] Behavioural & social sciences
- [ ] Ecological, evolutionary & environmental sciences

For a reference copy of the document with all sections, see nature.com/documents/nr-reporting-summary-flat.pdf

Life sciences study design

All studies must disclose on these points even when the disclosure is negative.

Sample size	For the cryo-EM data, we collected >5,000 raw movie micrographs using a Titan Krios electron microscope. We chose 382,478 raw particles for the rpl4Δ63-87 mutant and 332,847 raw particles for the nog1ΔC rei1ΔC reh1ΔC mutant. After 2D classification, all particles for the rpl4Δ63-87 mutant were used to produce clear 2D averages retained in the dataset. After 3D classification, the raw particles were applied for high-resolution refinement resulted in the final 3D maps of seven different classes. After 2D classification of the nog1ΔC rei1ΔC reh1ΔC mutant, 297,685 "good" particles that produced clear 2D averages were retained in the dataset. After 3D classification, the raw particles were applied for high-resolution refinement resulted in the final 3D maps of four major classes. The sample size was deemed sufficient because the data yielded our targeted resolution.
Data exclusions	Regarding the cryo-EM raw micrograph screening, exclusions were done based on the quality of the images and the presence of ice contamination. Regarding the particle selection, 2D and 3D classification were used, and criterion was based on the quality of resulting 2D class average and 3D maps. This criteria is empirical, but is a standard image processing practice in the cryo-EM community.
Replication	For the cryo-EM data, the reproducibility lies in a large number of particles used to derive final 3D maps. Reliability and resolution are measured by gold-standard Fourier shell correlation (Supplementary Figures 2c, 2d, 7c, and 8). The replication efforts through multiple refinement runs were successful and yielded similar 3D maps.<br>For all other experiments, there is a section in the Methods, titled “Statistics and Reproducibility”, stating the number of times each experiment was performed for relevant figures. All of our duplicated experiments are biological replicates, not technical replicates.
Randomization	The raw particles were randomly selected by a computer program (RELION 3.0). Cryo-EM reconstructions use two randomized half-sets to prevent over-refinement of the model, and to assess the resolution of the final model.
Blinding	Investigators were not blinded during grouping, since it is performed computationally.

Reporting for specific materials, systems and methods

We require information from authors about some types of materials, experimental systems and methods used in many studies. Here, indicate whether each material, system or method listed is relevant to your study. If you are not sure if a list item applies to your research, read the appropriate section before selecting a response.

Materials & experimental systems

n/a	Involved in the study	
[ ]	[x]	Antibodies
[x]	[ ]	Eukaryotic cell lines
[x]	[ ]	Palaeontology
[x]	[ ]	Animals and other organisms
[x]	[ ]	Human research participants
[x]	[ ]	Clinical data

Methods

n/a	Involved in the study	
[x]	[ ]	ChIP-seq
[x]	[ ]	Flow cytometry
[x]	[ ]	MRI-based neuroimaging

Antibodies

Antibodies used

Anti-Nog2 and anti-Tif6 were gifted to us by Cosmin Saveanu and Micheline Fromont-Racine (Institut Pasteur, France), respectively. Anti-Bud20 and anti-Nug1 were gifted to us by Vikram Panse (ETH Zürich, Germany). Anti-Nog1 was gifted to us by Janine Maddock (University of Michigan). Anti-uL4 was gifted to us by Lasse Lindahl (University of Maryland). Ant-Sda1 was gifted to us by Doug Kellogg (University of California Santa Cruz). All antibodies are polyclonal anti-rabbit.

Dilutions:

Sda1: 1:100

Bud20: 1:4,000

Nug1: 1:1,000

Nog2: 1:5,000

Nog1: 1:2,000

uL4: 1:1,000

Validation

Anti-Sda1 antibody:
Antibodies that recognize Sda1 were raised by immunizing rabbits with a COOH-terminal fragment of Sda1, purified from bacteria as a glutathione S-transferase (GST) fusion protein. The COOH-terminal fragment was amplified by PCR and cloned into the BamHI and EcoRI sites of pGEX-1 to create pZZ8, which expresses the COOHterminal fragment as a GST fusion. An identical fragment of Sda1 was cloned into the BamHI and EcoRI sites of pMAL-c2 to create pZZ6, which expresses the COOH-terminal fragment as an MBP fusion (New England Biolabs, Beverly, MA). Sda1 antibodies were affinity purified from serum by using the purifiedSda1-MBP fusion protein coupled to Affi-gel 10 (Bio-Rad Laboratories, Hercules, CA) as previously described (Kellogg and Alberts, 1992).
Reference: https://www.ncbi.nlm.nih.gov/pmc/articles/PMC30578/pdf/mk000201.pdf

Anti-Nog2 antibody:
Rabbit polyclonal Nog2-specific antibody was generated in Micheline Fromont-Racine's lab (Institut Pasteur, France) by using recombinant glutathione S-transferase (GST)-Nog2 purified from Escherichia coli.
Reference: http://doi.org/10.1128/MCB.23.13.4449-4460.2003

Anti-Bud20 antibody:
Recombinant anti-Bud20 was produced in BL21 E. coli strain by IPTG induction and affinity purified using Ni sepharose (GE Healthcare, Uppsala, Sweden).
Reference: https://www.ncbi.nlm.nih.gov/pmc/articles/PMC3542530/pdf/msb201263.pdf

Anti-Nug1 antibody:
Rabbit Polyclonal anti-Nug1 antibody was generated in Vikram G Panse's laboratory (Institute of Medical Microbiology, University of Zurich, Zurich, Switzerland).
Reference: file: https://doi.org/10.7554/eLife.52474

Anti-Nog1 antibody:
Antibodies were raised against HIS6Nog1 expressed from pJF14 in E. coli BL21 as previously described for anti-Mtg2 antibodies (Datta et al. 2005). The antibodies were affinity purified using HIS6Nog1 transferred to nitrocellulose, as previously described (Salamitou et al. 1994) before use.
Reference: https://doi.org/10.1007/s00438-007-0233-1

Anti-uL4 antibody:
Antigens for these were synthetic peptides corresponding to the first 20 amino acids ofE. coli uL4 and uL22, respectively. Antisera from rabbits used for the rest of the experiments were prepared for the Lindahl lab (University of Maryland) by Covance Research Products, Denver, CO, USA using synthetic pep- tides corresponding to the N-terminal 21 (uL4) or 23 (uL22) amino acids of the respective E. coli proteins.
Reference: https://www.ncbi.nlm.nih.gov/pmc/articles/PMC4937340/pdf/gkw493.pdf

IgG antibody product information:
Purified rabbit IgG is isolated from pooled normal rabbit serum by fractionation and ion-exchange chromatography. Purified rabbit IgG may be used as a reference antigen, standard, blocking agent, or coating protein in a variety of immunoassays including ELISA, dot immunobinding, western immunoblotting, immunodiffusion, and immunoelectrophoresis. Other applications include starting materials for the preparation of immunogens and solid phase immunoadsorbents.
In this manuscript, IgG was used for affinity purifications of preribosomes. Since the TAP tag contains protein A, any IgG can be used to affinity purify or detect TAP-tagged proteins..
https://www.sigmaaldrich.com/content/dam/sigma-aldrich/docs/Sigma/Product_Information_Sheet/2/i5006pis.pdf
