# Supplementary material for: Structural insights into assembly of the ribosomal nascent polypeptide exit tunnel
Source: Nat Commun. 2020 Oct 9;11:5111. doi: 10.1038/s41467-020-18878-8 (PMC7547690; doi:10.1038/s41467-020-18878-8)
Supplement: Supplementary file 2 — Peer Review [file 41467_2020_18878_MOESM2_ESM.pdf]

Reviewers' comments:

Reviewer #1 (Remarks to the Author):

Comments to Wilson et al., 2020:

The nascent polypeptide exit tunnel is an important functional element of the the ribosome. While the formation of the adjacent peptidyl transferase active site (the ribosomal catalytic centre) and the proofreading of its correct assembly have been extensively studied, the mechanisms that govern the assembly and possible proofreading of the tunnel, and how these processes are coordinated with 60S subunit assembly overall, are less well understood. In their important contribution, Wilson et al. now report novel and interesting data that provide insight into the the consequences of mutations of ribosomal protein L4 that forms part of the tunnel wall, and of ribosome assembly factors that insert extended tails into the tunnel. Using biochemistry and cryo-electron microscopy, they show that ribosome assembly is perturbed in these mutant cells, and that these perturbations may arise from aberrant conformations of ribosomal RNA segments in the vicinity of the peptidyl transferase centre.

The manuscript presented here adds valuable data and insight, and it aids our understanding of ribosome assembly. I recommend publication of the manuscript, under the condition that the comments below are satisfactorily addressed. Special attention should be given to proper validation of cryo-EM maps and models, and to deposition of all maps and coordinate models that are critical to the main points of the manuscript.

Specific comments:

The supplementary figure legends appear to be out of order. The legend "Supplementary Figure 4" seems to go with the last figure in the supplementary figures file.

Fig. 2b is referenced out of order.

Fig. 2f: Most of the changes seem to be similar between replicates. Can the authors comment on the exceptions to this observation (e.g. Nop53, Bud20)? Does this impact the reliability of the other measurements?

Page 11: How do the observed defects in the Nog1 CTD mutants compare to those observed for mutants in other NPET-inserting assembly factors?

Supplementary Fig. 5: Classes N2-N4 are shown only as 3D classification results, not as 3D-refined maps. These maps should be fully refined (as was done for N1). FSC curves for all classes displayed and interpreted in the manuscript (N1-N4) are required.

Supplementary Fig. 6: Weak density of Rix1/Rea1: Is this due to low occupancy or due to conformational heterogeneity of the associated factors? In the former case, further 3D classification should be able to recover the bound and unbound states. Is the cryo-EM particle box size chosen large enough to fully accommodate Rea1?

Structure validation: I have not found a reference to deposition of the cryo-EM maps or the refined models to the corresponding databases (EMDB and PDB, respectively). Additionally, while there is a description of how the atomic models were built and refined, there is no description of how the models were validated, there are no tables with refinement statistics for the atomic models, and Fourier Shell Correlation curves (model vs. map FSC) that demonstrate the fit of the atomic models to the maps are lacking. All of these elements (including accession codes for deposited maps and models) must be added to the manuscripts before publication. This is in addition to the comment on lack of fully refined

maps in Supplementary Fig. 5 above.

Minor comments:

Page 3 (and after): I would encourage the authors to use the unified nomenclature for ribosomal proteins (e.g. uL4, uL22 rather than L4, L17) throughout the manuscript.

Page 9: This section raises the question if Arx1 recruitment is impaired by the L4 mutation directly, or via Nog1. The authors later address this (Discussion), but adding a short statement (one sentence) on this topic to this section might be helpful to readers.

Page 9: R2 is described as a precursor to R1. What is this assessment based on? What are the other 60% of particles in this dataset (are they all earlier intermediates)?

Supplementary Fig. 4b: Could the authors add the 40S, 60S, and 80S peak labels to all UV traces? For the specialist reader, this information is easy to deduce, however, for a more general audience this may be helpful. Having the axes displayed for all traces may be helpful as well.

Reviewer #2 (Remarks to the Author):

Wilson et al, present the characterization of the Rpl4 Δ63-87 single and Nog1ΔC Rei1ΔC Reh1ΔC triple mutant. These deletions have been shown previously to be part of the PET (L4) or be positioned inside the nascent PET (terminal extension of Nog1, Rei1, Reh1). The authors have done a basic characterization of these mutants (growths phenotype, Rpl25-GFP localization, Sucrose gradient analysis) and most informative revealed cryo EM maps of nuclear pre-60S particles isolated from these mutant backgrounds.
The isolated pre-60S particles (via Nog2 bait protein) are characterized by the pre-rotated state of the 5S RNP and an improper orientation of the rRNA helices H75-79 and H68-69, which can be seen in the new cryo-EM structures.
Mass spectroscopy (ms) indicates that Rea1 and the Rix1 subcomplex is strongly reduced in these particles, which could be a result of the improper orientation of the rRNA helices H75-79 and H68-69. The authors conclude form these data that maturation of the PET is linked to rotation of the 5S RNP which depends on Rea1 and the Rix1 subcomplex.

However, this conclusion needs more validation. A recent Cryo-EM of Nog2 purified particle from wild-type background was also largely missing Rea1, Rix1 and Sda1. Thus, up to now only the ms data suggest a connection between NPET and 5S rotation. It is required that this is further confirmed by additional biochemical experiments. I suggest to do sucrose gradient analysis and determine the sedimentation of Rix1, Ipi1, Ipi3, Rea1, Sda1 by Western blot. If the conclusion is true, these factors should be shifted to the upper fractions of the gradient. Moreover, a purification of these factors should reveal a decreased association with pre-ribosomal particles.

Woolford and colleagues discuss that the improper orientation of rRNA helices H75-79 and H68-69 are responsible for the putative reduced recruitment of Sda1, Rix1 subcomplex, Rea1. This argument seems very plausible; however, the manuscript leaves open how the NPET affects the conformation of H75-79 and H68-69. The presented cryo-EM pictures (Figure 4) suggest that the basis of the RNA helices is correctly positioned and the NPET is correctly formed? Thus, it remains unclear why the tip of H75-79 and H68-69 should be affected? Here, a better discussion and better pictures are required.

Detailed Comments

1. In the introduction the protein L17, L39 are mentioned. In would be helpful to see the position of these proteins in Figure 1? It might be helpful to add the structure of a matured ribosome, where the L-proteins are highlighted.
2. Please label the rRNA helices in Figure 1
3. Several experiments are missing relevant information in the Figure legend. In the current state only, few experts will be able to follow the experimental procedures.
Figure 2a: Growth phenotype appears not fully clear, to clarify that please indicate which type of strain has been used (wt or L4 deletion) and the type of L4 plasmids (promotor?).
Figure 2C: Please give the incubation time of the cell in Glucose within the Figure legend.
Figure 2f, 5c: I find it very hard to interpret the presented data. Please explain in the figure legend how the numbers were calculated.

Fold change (B-A):A, Ratio B:A ? Log2 ?

… Please check all Figure legend for missing information.
4. Figure 2d: Localization of L25 eGFP is not a precise method to determine at which steps 60S assembly is blocked. I suggest to move this experiment to supplemental data. Additionally, it might be helpful to see the L25 localization within cells that carry the empty vector.
5. Figure 2e: Use Western blot analysis to monitor the L4 levels in the purified particles. Since mature ribosomes could be contaminates within the eluates, a Western blot signal give more information (shift in protein size) than a mass spec analysis. Please indicate whether a single step or tandem purification has been performed.
6. Figure 5b: Add Western blot analysis for relevant proteins.
7. The term NPET-inclusive sequences might be replaced by NPET-invasive sequences?
8. "This bulky GFP tag prevents insertion of the Nog1 CTD into the tunnel." Here, also other possibilities could be possible. Maybe, the NPET is formed around Nog1 CTD. In this case, insertion of the CTD might be fine, but the assembly around the CTD is disturbed

The ms needs a major revision before it can be consider for publication.

In response to reviewer #1.

The manuscript presented here adds valuable data and insight, and it aids our understanding of ribosome assembly. I recommend publication of the manuscript, under the condition that the comments below are satsifactorilly addressed. Special attention should be given to proper validation of cryo-EM maps and models, and to deposition of all maps and coordinates that are critical to the main points of the manuscript.

We thank the reviewer for appreciating our manuscript. We have substantially revised the text and modified the figures throughout the manuscript. The cryo-EM maps and molecular models have been deposited, and the accession numbers are provided in the revised version of the manuscript.

Specific comments:

The supplementary figure legends appear to be out of order. The legend "Supplementary Figure 4" seems to go with the last figure in the supplementary figures file.

Fig. 2b is referenced out of order.

We thank the reviewer for pointing out these discrepancies and have corrected them.

Fig. 2f: Most of the changes seem to be similar between replicates. Can the authors comment on the exceptions to this observation (e.g. Nop53, Bud20)? Does this impact the reliability of the other measurements?

We looked carefully at our mass-spec data for Nop53 and Bud20. We did not find any evidence of low peptide coverage or other anomalies that could explain the differences in levels of Nop53 and Bud20 in replicates. We suspect that the fraction of particles containing or missing an assembly factor like Nop53 or Bud20 can vary depending on subtle differences in the replicates. For example, slight differences in the OD at which the cells were harvested might result in minute differences in levels of some proteins. In order to strengthen our results, we have now added data for several other assembly factors, measured by western blotting and mass-spectrometry. We added western blotting data for Bud20 and Nug1 to Figure 2e, and for Nug1, Tif6, and Bud20 to Figure 5b. We added western blot data for Ecm1, Mrt4, Cic1, and Rlp7 to Figure 2f, and for Cic1, Mrt4, and Rlp7 to Figure 5c. These additions clearly show that results for most assembly factors were consistent among replicates and did not change in mutant strains relative to wild-type strains. We now also show a standard deviation statistical analysis across 3 replicates.

Importantly, the entry and exit of Nop53 and Bud20 are uncoupled from the assembly event blocked in our *rpl4* mutant (5S RNP rotation). Assembly of Nop53 helps

initiate ITS2 spacer removal, which is uncoupled from 5S RNP rotation (refs 23, 29). Bud20 is a nonessential AF known to be an export factor, but it is not yet clear what is the trigger for its entry into pre-ribosomes. What is very clear from our data, however, is that assembly factors necessary for 5S RNP rotation were consistently decreased in replicates, and levels of other assembly factors now added as controls were not changing. Therefore, we strongly believe that, regardless of whether changes in levels of Nop53 and Bud20 are of technical or biological nature, they are not useful or relevant to explain our phenotypes, and do not play a role in the block seen in our mutant strains.

Page 11: How do the observed defects in the Nog1 CTD mutants compare to those observed for mutants in other NPET-inserting assembly factors?

In our revised manuscript, we have added text to the Discussion section starting on page 17 line 5 to address this question. It reads, “Our results also suggest that Nog1 plays the most important role of these three AFs (Nog1, Rei1, and Reh1). Analogous C-terminal truncation of Rei1 has minimal effects on cell growth at all temperatures and *reh1*Δ cells have no obvious growth defect[22,28]. Nevertheless, our triple truncation mutant, *nog1ΔC rei1ΔC reh1ΔC,* did display a slightly slower growth rate at 30ºC than the *nog1Δ595-647* single mutant alone. This leaves the possibility that the NPET-occupying amino acids in the CTDs of either Rei1 or Reh1 perform a minor yet still unknown function.”

Therefore, Nog1 is the only factor to date whose tunnel-inserted portions are known to be important for growth. Note that longer truncations of Rei1 beyond the portion in the tunnel, and a complete deletion of *REI1* result in severe cold-sensitive growth defects. However, these defects were shown to be due to a failure to remove another assembly factor, Arx1, from assembling pre-60S subunits (refs. 22 and 39). We were mindful of this relationship between Arx1 and Rei1 and did not remove the Arx1-interacting helix from Rei1 in our triple truncation mutant (*nog1ΔC rei1ΔC reh1ΔC*). Because Arx1 is an assembly factor that binds outside the exit of the NPET, we consider this defect to be entirely separate from effects on tunnel construction that we have uncovered in this manuscript.

Furthermore, effects of mutations in the assembly factors that associate with the NPET before Nog1 (Rrp14, Rpf1, and Spb1), have not yet been examined, except for complete depletion of each factor, which block assembly very early and lead to rapid turnover of pre-ribosomes.

Supplementary Fig. 5: Classes N2-N4 are shown only as 3D classification results, not as 3D-refined maps. These maps should be fully refined (as was done for N1). FSC curves for all classes displayed and interpreted in the manuscript (N1-N4) are required.

We agree with the reviewer and have now performed 3D refinement for these classes. Relevant figures, including Supplementary Figures 1 and 8, have been updated.

Supplementary Fig. 6: Weak density of Rix1/Rea1: Is this due to low occupancy or due to conformational heterogeneity of the associated factors? In the former case, further 3D classification should be able to recover the bound and unbound states. Is the cryo-EM particle box size chosen large enough to fully accommodate Rea1?

We thank the reviewer for pointing this out. We have performed further 3D classification on this group of particles and were unable to split them. This suggests that these particles exhibit large conformational heterogeneity in the Rix1 region (as indicated by fragmented densities of the Rix1 complex in **Supplementary Fig. 6b**, which is now **Fig. 6d**). As for Rea1, its density is nearly completely missing in the map. We also tested a larger window size (400 pixels) but failed to get an improved density map for both factors.

We realized that our previous description of this class was not accurate enough. Therefore, we have changed the text on page 14 line 5 to "Unique to all mutant pre-60S particles presented thus far, class N4 contains a rotated 5S RNP and displays density for Sda1, identifying class N4 as a Nog2 state 2-like particle. However, in contrast to wild-type Nog2 state 2 where densities for the Rix1 complex and Rea1 are clearly visible, the densities for the Rix1 complex were weak and Rea1 was nearly completely missing in class N4 (**Fig. 6d**). Again, **Supplementary Fig. 6b** is now **Fig. 6d**.

Structure validation: I have not found a reference to deposition of the cryo-EM maps or the refined models to the corresponding databases (EMDB and PDB, respectively). Additionally, while there is a description of how the atomic models were built and refined, there is no description of how the models were validated, there are no tables with refinement statistics for the atomic models, and Fourier Shell Correlation curves (model vs. map FSC) that demonstrate the fit of the atomic models to the maps are lacking. All of these elements (including accession codes for deposited maps and models) must be added to the manuscripts before publication. This is in addition to the comment on lack of fully refined maps in Supplementary Fig. 5 above.

The cryo-EM maps and molecular models now have been deposited and accession numbers have been provided. In the revision, as requested by the reviewers, we have added relevant details of model validation in the Methods section (page 26, line 9). Refinement statistics of atomic models have been added to Supplementary Table 1. Also, we have added model versus map and model validation FSC curves to **Supplementary Figs. 1c and 1d.**

End of response to reviewer #1.

In response to reviewer #2.

Mass spectroscopy (ms) indicates that Rea1 and the Rix1 subcomplex is strongly reduced in these particles, which could be a result of the improper orientation of the rRNA helices H75-79 and H68-69. The authors conclude form these data that maturation of the PET is linked to rotation of the 5S RNP which depends on Rea1 and the Rix1 subcomplex.
However, this conclusion needs more validation. A recent Cryo-EM of Nog2 purified particle from wild-type background was also largely missing Rea1, Rix1 and Sda1. Thus, up to now only the ms data suggest a connection between NPET and 5S rotation. It is required that this is further confirmed by additional biochemical experiments. I suggest to do sucrose gradient analysis and determine the sedimentation of Rix1, Ipi1, Ipi3, Rea1, Sda1 by Western blot. If the conclusion is true, these factors should be shifted to the upper fractions of the gradient. Moreover, a purification of these factors should reveal a decreased association with pre-ribosomal particles.

We thank the reviewer for their critical thoughts and suggestions on how to make our argument stronger. However, in addition to mass-spec data, there are other data in this manuscript that suggest a connection between assembly of the NPET and 5S RNP rotation. Our conclusions are based on several complementary approaches: silver stained SDS-PAGE gels, western blotting, mass spectrometry and cryo-EM of affinity purified pre-ribosomes. Furthermore, our microscopy and pre-rRNA processing data are consistent with a block at this stage of 60S assembly. Of all of these approaches, cryo-EM is the best approach to determine the presence or absence of assembly factors in the pre-60S particles, in particular when wild-type controls are available. Previously, we purified particles associated with Nog2 from wild-type cells (ref. 17) and characterized the structures of three major classes of particles (Nog2 states 1-3). While it is true that Nog2 state 1 does not contain Rea1, the Rix1 complex, and Sda1, the Nog2 state 2 particles contain clear densities for all of these assembly factors, and in that state, 5S RNP is rotated. We used these wild-type Nog2 particles as controls for our purifications of Nog2 particles from the *rpl4Δ63-87* and *nog1ΔC rei1ΔC reh1ΔC* mutants. Thus, if rotation can occur in our mutants, we expect to see particles that resemble Nog2 state 2 particles, containing Rea1, the Rix1 complex, and Sda1, as well as state 3 particles, where these assembly factors are missing, but the 5S RNP is in a rotated position. However, we did not observe any such particles in any *rpl4Δ63-87* mutant classes and only one class that we now describe as “Nog2 state 2-like” from the *nog1ΔC rei1ΔC reh1ΔC* mutant (class N4).

We acknowledge that the manuscript previously had more text elaborating on classes R3-R7 from the *rpl4Δ63*-87 mutant and that classes R5 and R6 resembled “Arx1-like particles.” However, these particles are missing large portions of rRNA and we thus consider them to be turnover intermediates or bad particles that may be distracting to a general audience. Although, we point out here that these particles indicate that pre-60S subunits in the *rpl4Δ63*-87 mutant that pass Nog2 state 1, for whatever reason, are destined for swift turnover. Thus, based on these data, we

strongly believe that our conclusion about the defect in NPET being communicated to the 5S RNP rotation is very solid and strong.

The original version of this manuscript elaborated on classes R3-R7 from the *rpl4Δ63*-87 mutant (this part of the manuscript has now been deleted, for clarity and space). Interestingly, classes R5 and R6 resembled “Arx1-like particles”, which correspond to Nog2 state 3 particles. However, these particles are missing large portions of rRNA and are thus considered to be turnover intermediates. That indicates that, even if a portion of particles can pass the block caused by the absence of the TD of uL4, they are quickly turned over and do not complete the assembly pathway. This rapid turnover is another indication for the importance of the TD of uL4 for the proper assembly of the NPET.

In summary, based on all the above data, we strongly believe that our conclusion about the defect in NPET being communicated to the 5S RNP rotation is very solid and strong.

We added text to the revised manuscript to explain Nog2 states 1-3 to the reader in the Results beginning on page 7 line 17, and in the Discussion beginning on page 15 line 15.

In regard to the experiment involving western blots across a sucrose gradient, we have reason to believe that this experiment would not clarify our data any further. The Rix1 complex and Rea1 interact with pre-ribosomes transiently. Pre-60S particles bound to the Rix1 complex and Rea1 are difficult to visualize in high-resolution, indicating that these particles are indeed short-lived (refs. 17 and 24). Therefore, we would expect that the majority of each of these proteins would sediment at the top of the gradient (where free proteins and small complexes sediment) even under wild-type conditions. Small amounts of the Rix1-complex and Rea1 may or may not be detectable in the pre-60S fractions of the gradient under wild-type conditions (there are no published results for this experiment with these proteins, to the best of our knowledge).Thus, any small differences in amounts of the Rix1-complex or Rea1 bound to pre-60S subunits in wild-type vs. mutant samples might not be detectable. When components of the Rix1 complex or Rea1 are used as baits for affinity purifications of pre-60S particles, the number of proteins visualized by the SDS-page gel is lower compared to the same experiment where the bait is a protein that remains bound to pre-ribosomes for an extended period of time (ref. 24). In addition, since antibodies against these proteins are not available, this experiment would require epitope tagging each of these proteins in order to be able to detect them by western blotting.

Overall, we feel that the payoff from this experiment would be unremarkable.

Woolford and colleagues discuss that the improper orientation of rRNA helices H75-79 and H68-69 are responsible for the putative reduced recruitment of Sda1, Rix1 subcomplex, Rea1. This argument seems very plausible; however, the manuscript leaves open how the NPET affects the conformation of H75-79 and H68-69. The presented cryo-EM pictures (Figure 4) suggest that the basis of the RNA helices is correctly positioned and the NPET is correctly formed? Thus, it remains unclear why the tip of H75-79 and H68-69 should be affected? Here, a better discussion and better pictures are required

We appreciate the reviewer's comments here and apologize that this point was not made clearer. While H75 and H68 are major focal points of Fig. 4, we also observed that H74, a component of the NPET, is visible yet flexible relative to its wild-type counterpart. Thus, rRNA from the NPET is affected in the *rpl4∆63-87* mutant class R1 (Fig. 4b). We hope that the following text that we have added to the Results and Discussion sections helps to clarify these points for the reader.

Results section starting on page 9 line 10: "Further analysis of the atomic model of class R1 revealed how a misassembled NPET could cause these rRNA conformational changes and block rotation of the 5S RNP through a series of striking rRNA rearrangements. These aberrant shifts begin when H74, an rRNA component of the NPET in close proximity to the uL4 TD, is displaced ~4Å relative to the wild-type conformation."

Discussion section starting on page 15 line 5: "the most significant changes involve improper conformations of rRNA helices. In the wild-type particles, H74 is most likely stabilized by the TD of uL4 in the NPET. In class R1, the absence of the uL4 TD changes the conformation of H74, which is communicated to H75, causing it to adopt an aberrant conformation."

Minor comments:

Page 3 (and after): I would encourage the authors to use the unified nomenclature for ribosomal proteins (e.g. uL4, uL22 rather than L4, L17) throughout the manuscript.

We thank this reviewer for the suggestion and have changed the nomenclature for all ribosomal proteins.

Page 9: This section raises the question if Arx1 recruitment is impaired by the L4 mutation directly, or via Nog1. The authors later address this (Discussion), but adding a short statement (one sentence) on this topic to this section might be helpful to readers.

We agree that this is an important idea to raise earlier in the manuscript, as the reviewer recommended. We have mentioned this result earlier in the Results section with added text starting on page 10, line 15.

Page 9: R2 is described as a precursor to R1. What is this assessment based on? What are the other 60% of particles in this dataset (are they all earlier intermediates)?

We apologize that this point was unclear in the original manuscript. We have significantly altered the text to make this interpretation clearer to the reader. This added text begins in the Results section on page 8 line 19 and reads as follows: "We obtained 7 classes of particles, which we refer to as classes R1-R7 (**Supplementary Fig. 1a, 1b**). Classes, R3-R7 were either missing large portions of rRNA and thus appeared to be turnover intermediates or represent earlier assembly intermediates and thus will not be discussed in depth here (**Supplementary Fig. 7**). The two remaining classes of pre-ribosomes, R1 and R2, were solved at a resolution of 3.2Å and 3.3Å, and made up 27%

and 12% of the total particles analyzed, respectively (**Fig. 3a** and **Supplementary Fig. 1c, 1d**). Since both R1 and R2 contain a pre-rotated 5S RNP and the AFs Rpf2 and Rrs1, they could be considered to most closely resemble Nog2 state 1 particles. As expected, density for the deleted TD of uL4 could not be observed in either class. While class R2 could be considered wild-type-like (see below), class R1 was more abundant than R2 and uniquely displayed aberrant rRNA conformations. Specifically, rRNA helices (H) 75-76 and H68-69 were shifted relative to the native positions observed in class R2 (**Fig. 3b**)."
We hope that this revision and our added text introducing Nog2 states 1-3 help to make this interpretation stronger and clearer.

Supplementary Fig. 4b: Could the authors add the 40S, 60S, and 80S peak labels to all UV traces? For the specialist reader, this information is easy to deduce, however, for a more general audience this may be helpful. Having the axes displayed for all traces may be helpful as well.

Thank you. We have made these changes to improve the aesthetics and the clarity of this Figure.

Detailed Comments

1. In the introduction the protein L17, L39 are mentioned. In would be helpful to see the position of these proteins in Figure 1? It might be helpful to add the structure of a matured ribosome, where the L-proteins are highlighted.

We agree that this figure is improved by showing more of the relevant NPET-associated proteins. We have added L17 (uL22) and eL39 to Figure 1a. We have also added the tunnel in mature 60S subunits to both Figures 1a and 1b.

2. Please label the rRNA helices in Figure 1

We thank the reviewer for this comment. However, the NPET is made up of many different rRNA helices, or portions thereof. The complete list of helices is as follows: H23, H24, H25, H25a, H32, H35, H35a, H47, H49a, H50, H61, H64, H65, H74, H89, H90, and H93. Thus, we believe that labeling all helices in Figure 1 will quickly make this figure extremely busy and not as accessible to a general reading audience.

3. Several experiments are missing relevant information in the Figure legend. In the current state only, few experts will be able to follow the experimental procedures.
Figure 2a: Growth phenotype appears not fully clear, to clarify that please indicate which type of strain has been used (wt or L4 deletion) and the type of L4 plasmids (promotor?).
Figure 2C: Please give the incubation time of the cell in Glucose within the Figure legend.
Figure 2f, 5c: I find it very hard to interpret the presented data. Please explain in the figure legend how the numbers were calculated.

Fold change (B-A):A, Ratio B:A ? Log2 ?
… Please check all Figure legend for missing information.

We apologize to the reviewer for this lack of clarity and appreciate the insight in helping us make this paper more accessible to readers. We have addressed these concerns by adding labels in Fig. 2a, adding incubation times in the Fig 2c legend, and specifying that we use fold change comparing mutant to wild-type in the Figs. 2f and 5c legends. We now have also more extensively elaborated on our experimental procedures in the Methods section.

4. Figure 2d: Localization of L25 eGFP is not a precise method to determine at which steps 60S assembly is blocked. I suggest to move this experiment to supplemental data. Additionally, it might be helpful to see the L25 localization within cells that carry the empty vector.

We agree with the reviewer that subcellular localization by microscopy is not a precise method to determine at exactly which step ribosome assembly is blocked. We did this experiment primarily to confirm that assembly is blocked before nuclear export of pre-ribosomes. We believe that all of the experiments shown together in Fig. 2 give a complete picture of the defect, and therefore present a very strong case for where assembly is blocked. For that reason, we believe that keeping microscopy data in Fig. 2 is helpful for easier understanding of the defect by a broad readership.

We have now changed Fig. 2d by adding an empty vector panel and changing the red color to magenta so that the signals can be more easily visualized.

5. Figure 2e: Use Western blot analysis to monitor the L4 levels in the purified particles. Since mature ribosomes could be contaminates within the eluates, a Western blot signal give more information (shift in protein size) than a mass spec analysis. Please indicate whether a single step or tandem purification has been performed.

We agree with this assessment and have added a western blot showing the shift in size of uL4. We have significantly elaborated how this experiment is done in the Methods section and specified that this is a one-step purification.

6. Figure 5b: Add Western blot analysis for relevant proteins.

We have added western blots for Nog1 to show a shift in protein size, Nog2 as a loading control, and Nug1, Tif6, and Bud20 as extra data points.

7. The term NPET-inclusive sequences might be replaced by NPET-invasive sequences?

We have settled on the term “inserted” in this context. We think that “invasive” gives the impression that these sequences do not normally belong in the tunnel, which we do not want to convey.

8. “This bulky GFP tag prevents insertion of the Nog1 CTD into the tunnel.” Here, also other possibilities could be possible. Maybe, the NPET is formed around Nog1 CTD. In this case, insertion of the CTD might be fine, but the assembly around the CTD is disturbed

We had this exact concern when we started investigation of the Nog1 CTD. However, now that the structures of earlier assembly intermediates, namely Nsa1-associated particles, have been visualized at high resolution with cryo-EM (ref. 16), we can see that the tunnel does not form around the Nog1 CTD. Instead, the NPET begins to form around the NTD of AF Rpf1 during early stages of 60S subunit assembly. The CTD of Nog1 only enters the tunnel after most of the tunnel is formed, when Nog2 binds to pre-60S subunits (Figure 1a). Thus, the bulky GFP tag on the Nog1 CTD most probably prevents entry of Nog1-GFP into the narrow confines of the tunnel at this stage.

End of response to reviewer #2.

Reviewer #1 (Remarks to the Author):

My concerns have been adequately addressed and I recommend publication of the manuscript.

Below are minor comments and possible typos:

Page 3, line 4 - angstrom is spelled out instead of using the symbol

Page 7, lines 17-21 - Citations of the primary literature may need to be added in this paragraph.

Supplementary Figure 1c, d - the point at which the model vs. map FSC crosses the FSC = 0.5 threshold should be indicated in addition to the half-map FSC = 0.143 threshold. This resolution value should then be reported in Supplementary Table 1 instead of the different model-map CCs (which on their own are not very informative).

Supplementary table 1 - Typo: C-beta deviation (deciation); clash score is missing; add model vs map resolution at FSC = 0.5 (see above).

Reviewer #2 (Remarks to the Author):

Woolford and colleagues have improved their manuscript in many small points according to the reviewer's suggestions.

However, my main criticism was not sufficiently addressed: The link between PET maturation and 5S rotation is to my opinion not firmly established. I still feel that only the presented ms data suggest that conclusion. But, also here the ms data does not completely fit to the Westernblot. (The ms data of Figure 2f (enrichment of Nug1, Tif6) does not nicely fit to Westernblot in Fig 2e. MS data (Fig 5c, decrease of Nug1) versus Westernblot 5B (increase of Nug1).)

Woolford argues that the EM analyses further underline that conclusion, but for that EM analysis of wt and mutant particles have to be done side by side with statistical analysis. One has to rule out that the effect is due to the temperature shift, or others effects? Only 39% of the EM classes has been considered for the particles from the rpl4Δ63-87 mutant, the rest has been put aside (turnover intermediates and others).
Moreover, no Western blot analysis has been done for the proteins involved in 5S rotation, nor gradient analysis. Thus, I think the ms is not suitable for publication in a nature journal.

In response to reviewer #1

My concerns have been adequately addressed and I recommend publication of the manuscript.

Below are minor comments and possible typos:

Page 3, line 4 - angstrom is spelled out instead of using the symbol
Page 7, lines 17-21 - Citations of the primary literature may need to be added in this paragraph.
Supplementary Figure 1c, d - the point at which the model vs. map FSC crosses the FSC = 0.5 threshold should be indicated in addition to the half-map FSC = 0.143 threshold. This resolution value should then be reported in Supplementary Table 1 instead of the different model-map CCs (which on their own are not very informative).
Supplementary table 1 - Typo: C-beta deviation (deciation); clash score is missing; add model vs map resolution at FSC = 0.5 (see above).

We have corrected these typos and oversights. We have added the FSC=0.5 threshold to Supplementary Figure 2C and D. The model vs. map resolutions at FSC=0.5 and clash score are added to Supplementary Table 1. We thank the reviewer for helping us improve the quality of this manuscript.

In response to reviewer #2

Woolford and colleagues have improved their manuscript in many small points according to the reviewer's suggestions.

However, my main criticism was not sufficiently addressed: The link between PET maturation and 5S rotation is to my opinion not firmly established. I still feel that only the presented ms data suggest that conclusion.

We are grateful to the reviewer for this criticism. After careful consideration and re-analysis, which involved refinement of all cryo-EM data gathered from the *rpl4Δ63-87* mutant (classes R1-R7), we agree that we over-stated the relationship between 5S RNP rotation and NPET assembly. Even more important, this re-analysis enabled us to refocus our manuscript and make significant additional conclusions about how NPET construction affects assembly of pre-60S subunits. We believe that our new model presented in this manuscript is impactful, important, and timely for the progress of our field of ribosome assembly. We strongly feel that these changes address the valid concerns of the reviewer, and also more accurately represent our presented data.

Beginning in the Results section on page 8, line 1, we describe how our cryo-EM analysis of *rpl4Δ63-87* mutant pre-60S particles revealed that, rather than only being blocked in 5S RNP rotation, this mutant is stalled or blocked in at least three distinct

steps of ribosome assembly. The common underlying cause among all three of these blocks is an inability to stabilize rRNA helix 74, one of the last components of the NPET to fully mature. We have significantly improved our text, including the Title, Abstract, Results, and Discussion sections to reflect and emphasize this observation and its consequences.

Briefly, deletion of the TD of uL4 destabilizes the three-way junction of rRNA helices H75, H76, and H79, resulting in a failure to maintain a docked conformation of H68. These effects on rRNA helices appear to arise during at least three different stages of 60S subunit assembly. Overall, a misassembled NPET appears to re-direct ribosome assembly to alternate pathways, some of which terminate in dead-end intermediates destined for turnover (classes R5 and R7). These conclusions are summarized and illustrated in a model shown in Figure 8.

But, also here the ms data does not completely fit to the Westernblot. (The ms data of Figure 2f (enrichment of Nug1, Tif6) does not nicely fit to Westernblot in Fig 2e. MS data (Fig 5c, decrease of Nug1) versus Westernblot 5B (increase of Nug1).)

We agree that these discrepancies exist. However, they do not impact our conclusions and may be explained by technical details such as antibody sensitivity and peptide coverage. We favor a technical explanation because the phenotypes observed in both mutants are not consistent with a defect in assembling or releasing Nug1 or Tif6 into pre-60S subunits. Such defects would block assembly upstream or downstream, respectively, of what we observe.

The interval in assembly on which we are focusing in this manuscript involves states 1, 2, and 3 of the Nog2 particle: starting with the entry of Sda1, then assembly of the Rix1 complex and Rea1 (dependent on Sda1), followed by exit of Rsa4. Levels of Sda1, an assembly factor important for the proper formation of Nog2 state 2 particles, consistently decreased, as assayed by silver-stained SDS-PAGE gels, western blotting, and mass spectrometry. In addition, Sda1 was not visualized by cryo-EM analyses of *rpl4Δ63-87* particles. Furthermore, analysis of our *nog1ΔC rei1ΔC reh1ΔC* mutant revealed a consistent decrease in Rea1 assayed by silver-stained SDS-PAGE gels, mass-spectrometry, and cryo-EM. Therefore, all of the proteins that impact our conclusions behave very consistently across our assays, including our cryo-EM analysis.

We included proteins like Nug1 and Tif6 for the sake of transparency. If the reviewer would prefer that we omit data for these proteins for the sake of clarity, we can certainly do that.

Woolford argues that the EM analyses further underline that conclusion, but for that EM analysis of wt and mutant particles have to be done side by side with statistical analysis. One has to rule out that the effect is due to the temperature shift, or others effects?

This first point is in response to the comments concerning the addition of an EM analysis of wild-type pre-60S particles alongside our mutant particles. We think out that wild-type and mutant cryo-EM of pre-ribosomal particles do not necessarily need to be done side by side. Once a wild-type structure is published, mutant or new structures can be superimposed onto or otherwise compared to the wild-type counterpart. Please refer to the following examples: Figure 6 in Thoms et al., 2018; Figure 4b in Sarkar et al., 2017; Figure 3d in Lyumkis et al., 2014; Figure 5b in Razi et al., 2019; Figure 2a in Hunziker et al., 2019; and Figure 6b in Mitterer et al., 2019. Nevertheless, we acknowledge that we cannot completely rule out the possibility that cold temperature could cause the structural defects that we see in our *nog1ΔC rei1ΔC reh1ΔC* mutant. However, we did perform sucrose gradients, pre-rRNA assays, and mass-spectrometry analyses with both wild-type and *nog1ΔC rei1ΔC reh1ΔC* mutant samples side by side with both samples being grown at 16ºC. Notably, results from mass-spectrometry analysis of this mutant were consistent with changes observed in particles revealed by our cryo-EM structures. Additionally, it is reasonable to assume that, if these rRNA conformational changes occur in response to a shift to cold temperature rather than our mutations, then we would see measurable ribosome assembly defects by other assays of wild-type cells shifted to the cold. However, our sucrose gradient fractionation experiments did not find any discernible defect in ribosomal subunit production in wild-type cells shifted to the cold (Supplementary Figure 6b). Therefore, we believe that our observations are unlikely to reflect changes due to temperature alone.

This second point is in response to the comment concerning a statistical analysis of our EM data. For this response, we have provided a root-mean square deviation statistical analysis between class R1 of the *rpl4Δ63-87* mutant particle and the wild-type Nog2 state 1 (PDB: 3JCT) atomic models. This analysis, done in PyMol (see below) shows that the most affected rRNA nucleotides correspond to the NPET, helices 68, 69, 75, and 76, and helices 88-89. The image below shows this analysis and was done in PyMol. Darker and wider pink rRNA ribbons represent nucleotides that are significantly deviated from their wild-type positions in 3JCT. The affected rRNA helices cluster around the NPET, H68, and the three-way junction of H75, H76, and H79. The insert on the right shows the location of the TD of uL4, and its proximity to H74.

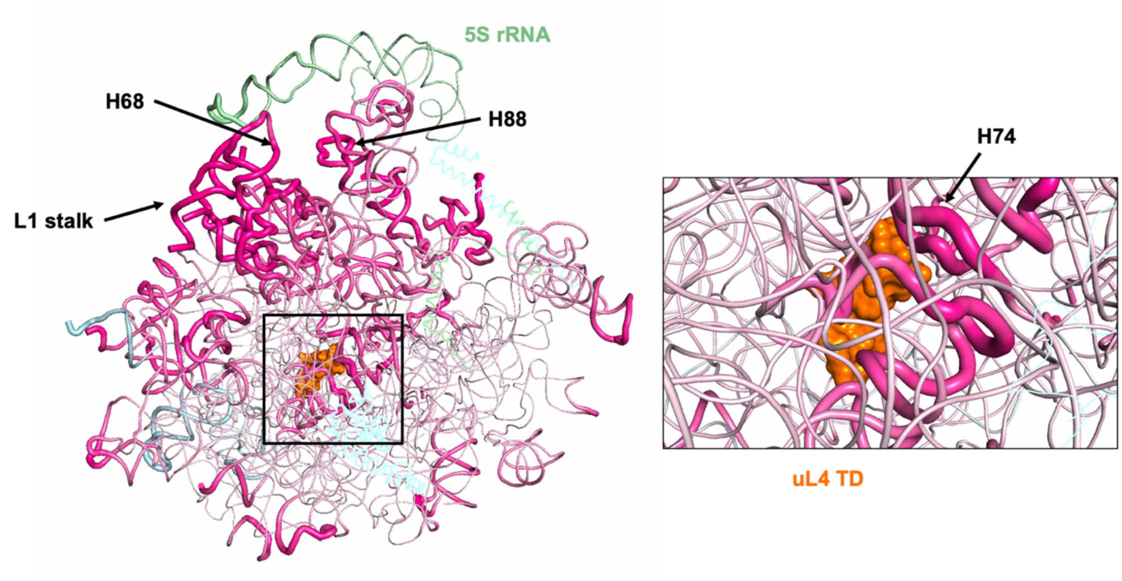

Only 39% of the EM classes has been considered for the particles from the rpl4∆63-87 mutant, the rest has been put aside (turnover intermediates and others).

Thank you for pointing us in a better direction. We have now included all particle classes from the *rpl4∆63-87* mutant in our description and interpretation of the data. Only two of these particle classes appear to be undergoing turnover (R5 and R7), as indicated by the lack of density for 25S rRNA domains III, IV, and V in class R5 and for domains III and IV in class R7. We believe that, by focusing on all classes of particles, instead only on the ones in which 5S RNP rotation is blocked, we have uncovered more principles of NPET assembly and painted a more complete picture of this stage of 60S subunit biogenesis.

Moreover, no Western blot analysis has been done for the proteins involved in 5S rotation, nor gradient analysis. Thus, I think the ms is not suitable for publication in a nature journal.

Because our manuscript now focuses on the assembly of the NPET and stabilization of rRNA, rather than 5S RNP rotation, we feel that these experiments are not going to improve the quality of the manuscript. Furthermore, we strongly believe that mass-spectrometry and cryo-EM are more sensitive and sophisticated assays than western blotting or sucrose gradient fractionation. There are currently two publications assessing mutations that affect association of the Rix1 complex and Rea1 to the pre-60S subunit (Figure 6d and Supplementary Figure 7b in Barrio-Garcia *et al*., 2015; and Figure 6 in Thoms *et al*., 2018). In both cases, cryo-EM was the gold standard for determining whether or not these proteins were able to associate with the pre-60S subunit and stabilize the 5S RNP in its rotated state. Neither westerns nor gradient analyses were performed on proteins required for 5S RNP rotation in order to support their interpretations. Additionally, Micic *et al.,* work from our lab that studied the same interval examined in this manuscript, was recently accepted by *Nature Communications.* This

manuscript made conclusions about the entry of the Rix1 complex and Rea1 into pre-60S subunits based on mass-spectrometry and cryo-EM and did not include western blots for these proteins. Therefore, we believe that our data are up to the highest standards in our field.

Figure 6 from Thoms et al., 2018, *Nature Communications*

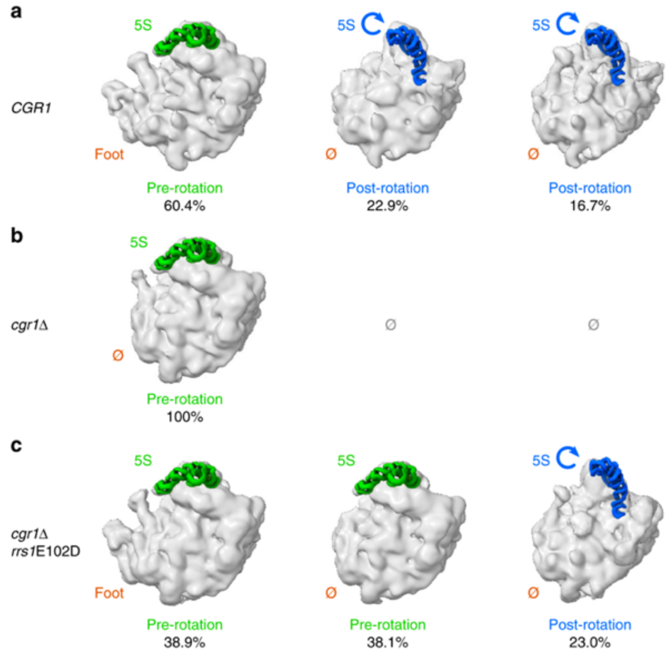

Supplementary figure 7b from Barrio-Garcia et al., 2015, *Nature Structural and Molecular Biology*

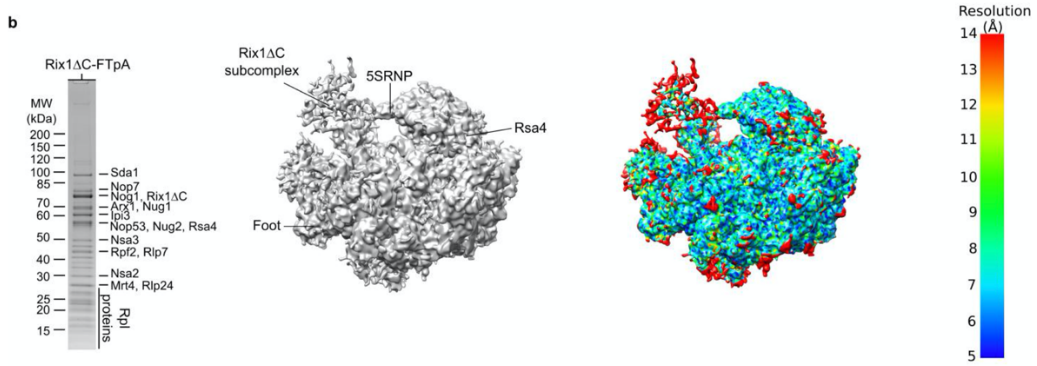

References

1. Thoms, M. *et al.* Suppressor mutations in Rpf2–Rrs1 or Rpl5 bypass the Cgr1 function for pre-ribosomal 5S RNP-rotation. *Nat. Commun.* **9**, 1–13 (2018).
2. Sarkar, A. *et al.* Preribosomes escaping from the nucleus are caught during translation by cytoplasmic quality control. *Nat. Struct. Mol. Biol.* **24**, 1107–1115 (2017).
3. Lyumkis, D. *et al.* Structural basis for translational surveillance by the large ribosomal subunit-associated protein quality control complex. *Proc. Natl. Acad.*

Sci. **111**, 15981–15986 (2014).
4. Razi, A. *et al.* Role of Era in assembly and homeostasis of the ribosomal small subunit. *Nucleic Acids Res.* **47**, 8301–8317 (2019).
5. Mitterer, V. *et al.* Conformational proofreading of distant 40S ribosomal subunit maturation events by a long-range communication mechanism. *Nat. Commun.* **2754**, (10AD).
6. Hunziker, M. *et al.* Conformational switches control early maturation of the eukaryotic small ribosomal subunit. *Elife* **8**, 1–16 (2019).
